# The association between anticholinergic burden and mobility: a systematic review and meta-analyses

**DOI:** 10.1186/s12877-023-03820-6

**Published:** 2023-03-22

**Authors:** Geofrey O. Phutietsile, Nikoletta Fotaki, Hamish A. Jamieson, Prasad S. Nishtala

**Affiliations:** 1 Department of Pharmacy and Pharmacology, University of Bath, Bath, BA2 7AY UK; 2 Centre for Therapeutic Innovation, University of Bath, Bath, BA2 7AY UK; 3 Department of Medicine, University of Otago, Christchurch, New Zealand

**Keywords:** Anticholinergic burden, Antimuscarinic, Physical function, Gait, Polypharmacy, Elderly

## Abstract

**Background:**

As people age, they accumulate several health conditions, requiring the use of multiple medications (polypharmacy) to treat them. One of the challenges with polypharmacy is the associated increase in anticholinergic exposure to older adults. In addition, several studies suggest an association between anticholinergic burden and declining physical function in older adults.

**Objective/Purpose:**

This systematic review aimed to synthesise data from published studies regarding the association between anticholinergic burden and mobility. The studies were critically appraised for the strength of their evidence.

**Methods:**

A systematic literature search was conducted across five electronic databases, EMBASE, CINAHL, PSYCHINFO, Cochrane CENTRAL and MEDLINE, from inception to December 2021, to identify studies on the association of anticholinergic burden with mobility. The search was performed following a strategy that converted concepts in the PECO elements into search terms, focusing on terms most likely to be found in the title and abstracts of the studies. For observational studies, the risk of bias was assessed using the Newcastle Ottawa Scale, and the Cochrane risk of bias tool was used for randomised trials. The GRADE criteria was used to rate confidence in evidence and conclusions. For the meta-analyses, we explored the heterogeneity using the Q test and I^2^ test and the publication bias using the funnel plot and Egger’s regression test. The meta-analyses were performed using Jeffreys’s Amazing Statistics Program (JASP).

**Results:**

Sixteen studies satisfied the inclusion criteria from an initial 496 studies. Fifteen studies identified a significant negative association of anticholinergic burden with mobility measures. One study did not find an association between anticholinergic intervention and mobility measures. Five studies included in the meta-analyses showed that anticholinergic burden significantly decreased walking speed (0.079 m/s ± 0.035 MD ± SE,95% CI: 0.010 to 0.149, *p* = 0.026), whilst a meta-analysis of four studies showed that anticholinergic burden significantly decreased physical function as measured by three variations of the Instrumental Activities of Daily Living (IADL) instrument 0.27 ± 0.12 (SMD ± SE,95% CI: 0.03 to 0.52), *p* = 0.027. The results of both meta-analyses had an I^2^ statistic of 99% for study heterogeneity. Egger’s test did not reveal publication bias.

**Conclusion:**

There is consensus in published literature suggesting a clear association between anticholinergic burden and mobility. Consideration of cognitive anticholinergic effects may be important in interpreting results regarding the association of anticholinergic burden and mobility as anticholinergic drugs may affect mobility through cognitive effects.

**Supplementary Information:**

The online version contains supplementary material available at 10.1186/s12877-023-03820-6.

## Background/Introduction

With increasing age comes age-related comorbidities that may be influenced by lifestyle, genomic makeup and other demographic factors. The increasing number of health issues require multiple medications (polypharmacy) to treat them. A 2005 study found that as of 2002, older adults defined as ≥ 65 years comprised 12% of the population of the United States but constituted 33% of its prescription drug expenditure (50 billion dollars) [1]. Whilst polypharmacy may be beneficial in treating underlying health conditions in older adults, it increases the risk of adverse drug events. In particular, taking multiple drugs with anticholinergic effects increases the risk of anticholinergic burden (AB) in older adults because of age-related pharmacokinetic and pharmacodynamic changes [2].

Mobility, defined as the ability to move independently in one’s environment, is crucial for independent living and good quality of life [3]. Declining mobility in older adults increases their dependence on other people to carry out basic activities of daily living. For example, the older adult or their family might require hiring a home carer or paying for care homes, which would have economic impacts. In countries with a free healthcare system, mobility status is a key determinant for accessing publicly funded healthcare support. Mobility decline in older adults thus has an economic impact on public finance and healthcare needs [4]. Mobility is multidimensional, and several tools may be utilised to obtain information about the mobility status of individuals. The methods assess gait, transfer skills, and activities of daily living (ADL) [5–7], among others.

The associations between anticholinergic burden and mobility have been found using AB scales to measure anticholinergic exposure [8] and have inspired intervention studies to determine ways to ameliorate the supposed negative association between AB and mobility. Squires et al. [9] investigated whether two different interventions could negate the association between AB and adverse effects such as mobility. In the study, AB was quantified using one of the several AB scales available in the literature. AB scales are tools designed to measure anticholinergic exposure, the majority of which exist in the form of scales that weigh each drug for its AB. A summary and comparison of commonly used AB scales can be found in the systematic review by Welsh et al. [10] and include; Anticholinergic Cognitive Burden (ACB), Anticholinergic Effect on Cognition (AEC), Anticholinergic Risk Scale (ARS), Anticholinergic Drug Scale (ADS), Serum Anticholinergic Activity (SAA) etc. The output of some of these scales has been correlated with health outcomes. For example, the SAA method, which measures the AB found in a serum sample, has been shown to predict the slowing of gait speed [11]. Other methods utilise expert opinion and binding affinity data [8] to rank drugs for AB potency. These scales have been shown to predict anticholinergic effects such as dizziness and constipation [12]. Predicting AB effects makes AB scales an important prognostic factor for impaired mobility.

This systematic review will synthesise data from published studies regarding the possible association between AB and mobility. In addition, the review will examine consistency in study findings to summarise the outcome of available primary research and thus better answer the question regarding the possible association between AB and mobility. A similar study by Mehdizadeh et al. [13] investigated the association of AB and mobility but for older people living with frailty [13], where the inclusion criteria included a requirement to have at least one measure to ascertain frailty. The study did not find an association between a one-unit increase in AB measured by the drug burden index and grip strength. Frail older adults have reduced reserves and thus may already have poor mobility [14]. Thus a study with only frail older people might suffer from the floor effect [15], in which any association between AB and mobility might be negligible because the participants already have impaired mobility.

## Objectives

### Primary objective

The primary objective is to investigate the association between AB and mobility.

### Secondary objectives

- To investigate if the association between AB and mobility differs based on the scales used to measure AB.
- To investigate a dose–response relationship between AB and mobility.
- To assess a threshold for AB that negatively impacts mobility.

## Methods

This systematic review and meta-analyses were conducted following guidelines from the Cochrane Handbook for Systematic Reviews of Interventions and the Preferred Reporting Items for Systematic Reviews and Meta-Analyses (PRISMA). In addition, the study protocol was registered with PROSPERO (CRD 42022301529).

### Data sources and search strategy

The search strategy was developed in collaboration with a librarian specialising in life sciences to explore search options and strategies. GP performed a literature search on five electronic databases; EMBASE, CINAHL, PSYCHINFO, Cochrane CENTRAL and MEDLINE. The search was conducted following a strategy that involved turning concepts in the PECO elements into search terms. The search timeline was for any studies published between January 1945-December 2021.

Medical Subject Headings (MeSH) were referred to optimise the best search terms to ensure that the searches capture synonyms of the same subject. The search terms were used with Boolean (AND/OR) to search for the most relevant publications. The following line of search terms was used to search across all the four electronic databases:

(“Antimuscarinic” OR “Anticholinergic”) AND (“Physical Function” OR “Grip” OR “Performance” OR “IADL” OR “SPPB”) AND (“elderly” OR “old” OR “geriatric”).

The terms were searched for in the title and abstract fields, with searches limited to observational studies, including case–control, cohort, pre-post intervention studies, and randomised control trials. The full search strategy is described in Additional File 1.

The search results of each database were then exported to EndNote reference managing software, where they were all combined and duplicates removed. From EndNote, the studies were exported to Covidence software [16] to exclude duplicates missed by EndNote before moving on to the next stage.

### Inclusion criteria

#### Study design

The inclusion criteria for the study designs that could be considered includes observational studies such as cross-sectional and cohort studies and experimental studies with randomised control trials.

#### Type of participants

The review only considered studies with older adults (≥ 65 years) or those in which all the participants were ≥ 65 years by the end of the study. We included participants from all settings, including, but not limited to, community-dwelling, hospital, specialist settings and those living in long-term care facilities. We did not exclude participants that did not have assessed mobility at baseline.

#### Type/s of intervention

The intervention in these studies was administering drugs with known or suspected anticholinergic properties, and all the medications considered appear in the composite reference scale of anticholinergic medicine [8].

#### Comparators

The outcome measures of patients with AB were compared with a control group with no AB and for patients with low AB versus those with high AB.

#### Outcomes

The outcome measure was functional mobility as measured by mobility scales such as the Barthel index (BI), activities of daily living (ADL) and instrumental activities of daily living (IADL). A comprehensive description of some mobility scales can be found in the systematic review by Soubra et al. [17], which reviews 31 assessment tests to evaluate mobility.

### Study selection

After removing duplicates, GP used Covidence software to screen the title and abstracts for inclusion or exclusion following an inclusion/exclusion criterion set out in the protocol document of this review. The software automatically highlighted inclusion and exclusion terms, allowing for easy screening. Studies without comparators were rejected, as well as systematic reviews or “review of reviews.” Participants in the study had to be $\ge 65$ years. In addition, the studies had to quantify anticholinergic medication exposure and have clinical outcome measures of mobility such as handgrip strength, gait speed, activities of daily living and other measures of physical function. Only studies that met the above criteria gleaned from the title and abstracts progressed to full-text screening and data extraction. Finally, full-text pdf copies of the studies were obtained for full-text analysis of the potentially eligible studies.

### Data extraction and analysis

Three researchers designed the data extraction sheet (GP, PN and NK). Information to be captured in the data extraction sheet involved the type of study, participant information, the anticholinergic medicines used, the anticholinergic scale used, methods of comparison, the types of outcome measures and whether the studies found a significant association between mobility and anticholinergic burden as given by the scales. In addition, sample size-dependent measures, such as setting p-value significance, were used as reported in the original studies. Finally, the Grades of Recommendation, Assessment, Development and Evaluation (GRADE) were used to assess the overall certainty of the evidence [18] (see Additional File 4 for GRADE evidence ratings. GRADE is a tool recommended by the Cochrane Collaboration to assess the evidence quality as presented by a systematic review’s conclusions [19, 20].

#### Risk of bias assessment

The Newcastle–Ottawa scale (NOS) was used to assess the risk of bias for cohort studies, and a modified version of it was used to assess cross-sectional studies [21, 22]. Studies that scored 6 or more points on the NOS were considered to have a low risk of bias given that they scored at least 3 points in the selection domain, one or more on the compatibility section and a minimum of 2 on the outcome domain. A moderate bias classification was assigned if the study scored 6 or more points but failed to meet one or more criteria for “low risk” classification. In contrast, a high risk of bias was assigned for studies that scored 5 or less for their overall NOS assessment. The Cochrane risk of bias tool for randomised trials was used (ROB1) [23] to assess the risk of bias for randomised control studies.

Heterogeneity among studies included for meta-analysis was quantified using Higgins and Thompson’s I^2^ statistic [24], with the following rough guide used to interpret the I^2^ statistic:

- 0 to 40%: might not be important
- 30 to 60%: may represent moderate heterogeneity
- 50 to 90%: may represent substantial heterogeneity
- 75 to 100%: considerable heterogeneity

A *p*-value threshold of 0.05 was used for statistical significance, with a *p* < 0.05 result considered statistically significant. Publication bias was assessed using a funnel plot [25], and Egger’s regression test was used to quantify the asymmetry of the plot [26]. The DerSimonian and Laird method [27] was used in the random-effects model to meta-analyse the studies. The meta-analyses were performed using JASP software [28] and Microsoft excel. Potential sources of heterogeneity across all studies were assessed using univariate meta-regression. Potential sources studied include 1)Continuous variable; mean age, percentage female 2)Categorical variable: AB scale used. The meta-regressions were performed using the Comprehensive Meta-Analysis (CMA) software [29].

## Results

### Study identification

**Fig. 1:**
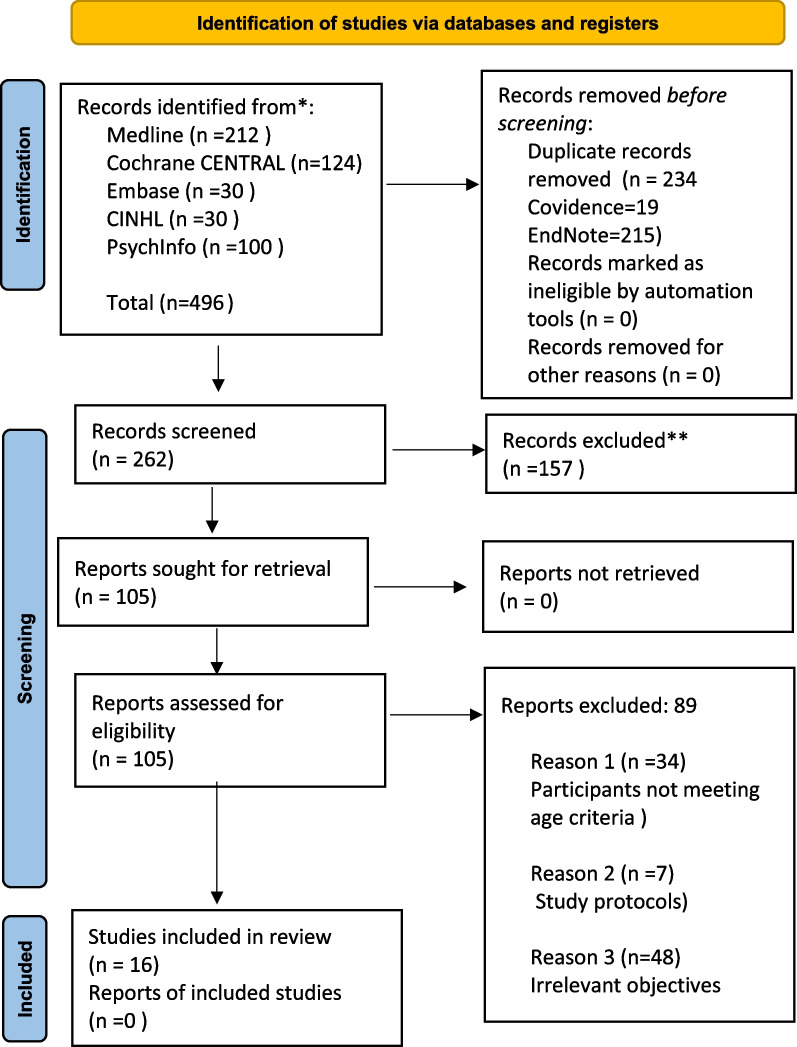
A PRISMA flow diagram demonstrating study selection, reasons for exclusion and number of studies included

Four hundred ninety six studies were obtained from searching the five databases: 212 from Medline, 124 from Cochrane CENTRAL, 100 from PsychInfo, 30 from Embase and 30 from CINAHL. EndNote software identified and removed 215 duplicates, leaving 281 studies. 19 more duplicates were identified by Covidence software and removed (including study protocols), leaving 262 studies for the title and abstract screening (Fig. 1). A re-run of the search strategy carried out before conducting the final analysis did not reveal any novel primary studies directly relevant to this research question. The re-run was carried out to consider any new primary studies whose results could have a bearing on the outcomes of this review.

#### Characteristics of included studies

The total number of included studies was *n* = 16, and all participants were ≥ 65 years. Of the 16 studies, five were included in the meta-analysis for the effects of AB on walking speed [11, 30–33], and four were included in the meta-analysis for the effects of AB on IADL scores [30, 32–34]. The included studies were a mixture of randomised control trials, and observational studies (commencement GRADE rating of moderate evidence of certainty). The most common type of studies were cross-sectional studies (*n* = 8), followed by cohort studies (*n* = 5) [31, 34–37] and only three were randomised controlled studies (*n* = 3) [38–40]. The studies used a mixture of statistical tests, but the t-test and *p*-value were the most commonly reported. A concise summary of study characteristics is presented in Table 1, and a brief description of some of the characteristics follows.

**Table 1:** Main characteristics of the 16 included studies on anticholinergic burden association with mobility measures

Author, Year	Country	Method	Study Setting	Anticholinergic Burden Scale Used	Mobility measures	Sample size	Association outcome
Soytas, 2021 [32]	Turkey	Cross-sectional (single centre)	Outpatient	ACB	BADL, HGS	256	Yes
Wouters, 2020 [31]	Netherlands	Prospective cohort study	Community dwelling	DBI	Walking test, Cardigan test, Chair Stand test, Balance Test, Functional Independence	3107	Yes
Attoh-Mensah, 2020 [41]	Switzerland	Cross-sectional	Community dwelling	ADS	Timed-Up and Go (TUG)	177	Yes
Mayer, 2017 [42]	Germany	Cross-sectional	Home dwelling	ABC, ACB, ADS, AAS, CrAS, ARS, ACL, Martindale, Cancelli	Barthel Index	2761	Yes
Kolanowski, 2015 [38]	USA	Randomized clinical trial	Hospital setting	ACB	Barthel Index	99	Yes
Landi, 2014 [35]	Italy	Prospective (Multicentre) Cohort	Nursing Homes	ARS	ADL	1490	Yes
Pasina, 2013 [43]	Italy	Cross sectional (prospective)	Hospital setting	ARS, ACB	Barthel Index	1380	Yes
Wilson, 2010 [39]	Australia	Randomized controlled trial	Residential Aged Care Facility	DBI	HGS, Walking speed, balance	602	No
Hilmer, 2009 [37]	USA	Prospective cohort	Community dwelling	DBI	SPPB, HGS	3075	Yes
Han, 2008 [34]	USA	Prospective Cohort	Community dwelling	CrAS	IADL	544	Yes
Gnjidic, 2008 [33]	Australia	Cross-sectional	Community dwelling	DBI	Performance Battery, HGS, IADL	1705	Yes
Cao, 2008 [44]	USA	Cross-sectional	Community dwelling	DBI	Balance, Gait speed, HGS, ADL, Mobility, Chair stand test	932	Yes
Nebes 2007 [11]	USA	Cross-sectional	Community dwelling	SAA	Gait, Manual response time	90	Yes
Landi, 2006 [30]	Italy	Cross-sectional cohort	Community dwelling	SAA	SPPB, HGS, IADL, ADL	364	Yes
Moretti, 2005 [36]	Italy	Controlled open-label study(Cohort)	Nursing Home	Olanzapine Vs -Promazine chloridate -Haloperidol	Barthel Index, IADL, Tinetti Scale	356	Yes
Street, 2000 [40]	USA	Randomised controlled trial (double-blind, placebo)	Nursing home	Olanzapine VS Placebo	Gait (Simpson Angus Scale Assessment)	206 (7 gait assessment)	Yes

KEY: *Cr-AS* clinician-rated anticholinergic scale, *DBI* drug burden index, *SAA* serum anticholinergic activity, *ACB* anticholinergic cognitive burden, *ARS* anticholinergic risk scale, *ADS* anticholinergic drug scale, *ADL* activities of daily living, *HGS* hand grip strength, *SPPB* short physical performance battery, *IADL* instrumental activities of daily living

##### Participant characteristics

Two studies included men only [33, 34], one study included women only [44], and the rest included both men and women. The study populations were distributed in four countries, with the USA being the most frequent (*n* = 6), followed by Italy (*n* = 4), Australia (*n* = 2), Turkey (*n* = 1), Netherlands (*n* = 1), Switzerland (*n* = 1) and Germany (*n* = 1) (Table 1). The study participants were mostly living in the community [11, 31, 33, 37], some were outpatients [32, 42], whilst others were based in a nursing home [35, 39]. The inclusion criteria of other studies were morbidity specific; People with Alzheimer’s [40], vascular dementia [36], hypertension [1], moderate to severe disability [44], as well as those with a history of falls [41].

##### Exposure (anticholinergic burden)

Except for two [36, 40], all studies used AB scales to measure exposure. The drug burden index (*n* = 5) was the most commonly used scale, followed by the anticholinergic cognitive burden scale (*n* = 4). Two studies used more than one AB scale to compare exposure as captured by the scales [42, 43].

##### Outcome measures

The primary outcome measure (mobility) was measured using several tools, including the Barthel Index [45], Timed-Up and Go (TUG) [46], handgrip strength [47], Basic Activities of Daily Living (BADL) [48], Gait assessment [49] etc. The most popular were handgrip strength and gait (n = 5). Ten of the studies used more than one mobility measure tool.

##### Risk of bias in included studies

The Newcastle–Ottawa Scale (NOS) [21] for assessing cohort studies was slightly modified and adapted for this systematic review and employed to assess the risk of bias for cohort studies (Table 2A) and cross-sectional studies (Table 2B). The modified scales are shown in the additional files (Additional files 2 and 5), elaborating on each assessment element and where each evidence can be found in each study. The cross-sectional NOS was used in preference to the newer appraisal tool for cross-sectional studies (AXIS) [50] as it has been shown to require considerably less time to complete and performs a methodological quality assessment to the same quality as the AXIS tool [51]. The cohort study by Landi et al. [35] had a moderate risk of bias because of scoring zero in the compatibility section, whilst all other cohort studies had a low risk of bias. The cross-sectional study by Soytas et al. [32] had a high risk of bias because of scoring an overall NOS score of 5, while all other cross-sectional studies had a low risk of bias. For the randomised control studies (*n* = 3), the Cochrane risk of bias tool for randomised trials was used (ROB1) [23]. The randomised control trial by Wilson et al. [39] had high-performance bias because no blinding among participants and research staff was carried out. The study also had high attrition bias because the adherence rate of participants had dropped to 46% by the end of the study. The ROB1 tool can be found in additional file 3 and is summarised in Table 2C. Overall, there was no serious concern regarding the risk of bias in the included studies, so this did not affect confidence in the certainty of the evidence. The three RTC’s were assigned on overall low risk of bias.

**Table 2:**
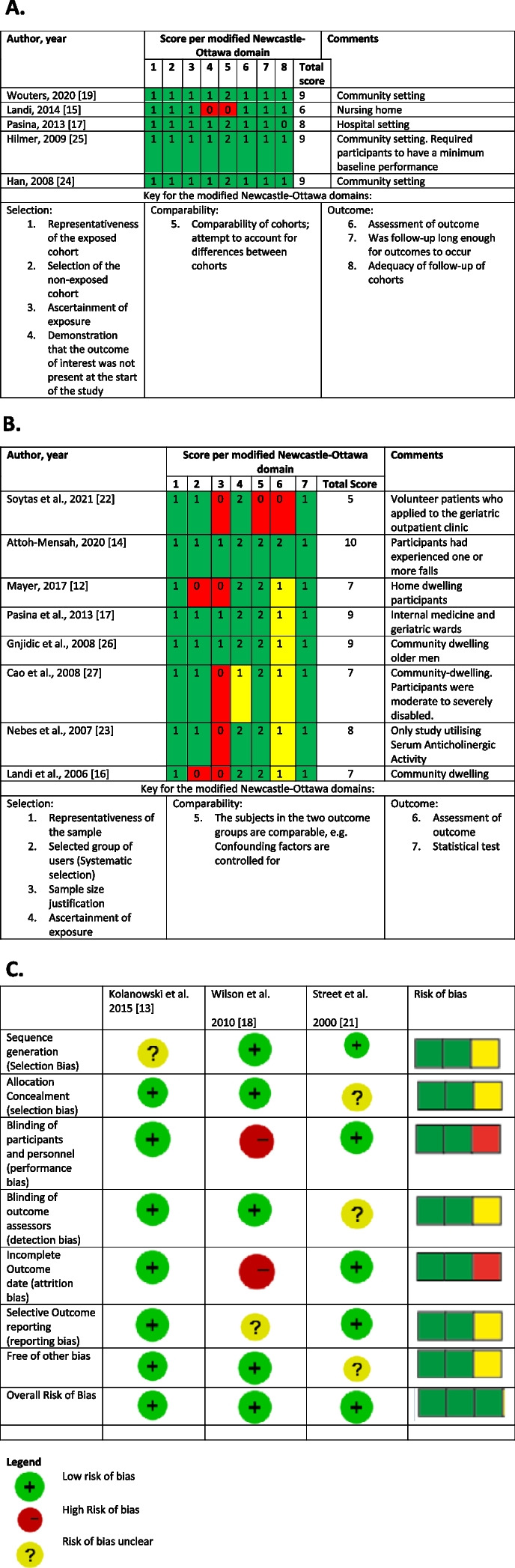
A) A summary of the quality assessment for cohort studies using a modified version of the Newcastle-Ottawa scoring system (summarised from Additional file 5). B) A summary of the quality assessment for Cross-Sectional studies using a modified version of the Newcastle-Ottawa scoring system adapted for cross-sectional studies. C) A summary of the Cochrane Collaboration’s tool for assessing risk of bias

A) Additional file 5, Wouters, 2020 [31], Landi, 2014 [35], Pasina, 2013 [43], Hilmer, 2009 [37], Han, 2008 [34]

B) Soytas et al., 2021 [32], Attoh-Mensah, 2020 [41], Mayer, 2017 [42], Pasina et al., 2013 [43], Gnjidic et al., 2008 [33], Cao et al., 2008 [44], Nebes et al., 2007 [11], Landi et al., 2006 [30]

C) Kolanowski et al. 2015 [38], Wilson et al. 2010 [39], Street et al. 2000 [40]

### Association between anticholinergic burden and mobility

Of the 16 studies, 15 demonstrated a statistically significant association between increasing anticholinergic burden and mobility [39]. For example, the paper by Soytas et al. [32] found that AB, as measured by the anticholinergic cognitive burden scale, decreased the handgrip strength in the exposure group compared to non-users (*p* = 0.04), whilst the difference in BADL performance was insignificant between the groups (*p* = 0.232). Nebes et al. initially found significant differences in all six mobility measures between users and non-users; however, this significance was lost in five of the six measures after adjusting for the effects of confounding factors [11], with hand grip strength retaining significance. For conducting meta-analysis, only two studies [30, 35] provided enough information on ADL measurements to conduct a meta-analysis of the association between AB and ADL, whilst one study [41] provided enough information on TUG measurements to conduct a meta-analysis on the association between AB and TUG. Thus the number of studies was too small to conduct a meaningful analysis of these outcomes. The same argument applied to studies with enough information on SPPB scores [30] and BADL [32]. Conversely, five studies provided enough information for a meta-analysis of the association between AB and walking speed [11, 30–33] to be conducted and four studies [30, 32–34] provided enough information for a meta-analysis of the association between AB and IADL to be conducted, and these form the basis of the two meta-analyses conducted in this study. Although the study by Moretti et al. [36] provided enough information regarding IADL scores, it was not included in the meta-analysis of IADL and AB because instead of having a control group like the other four IADL studies, the study had participants using haloperidol; a less potent anticholinergic drug, and compared the group with participants taking olanzapine; a more potent anticholinergic drug.

### Association between anticholinergic burden and walking speed

**Fig. 2:**
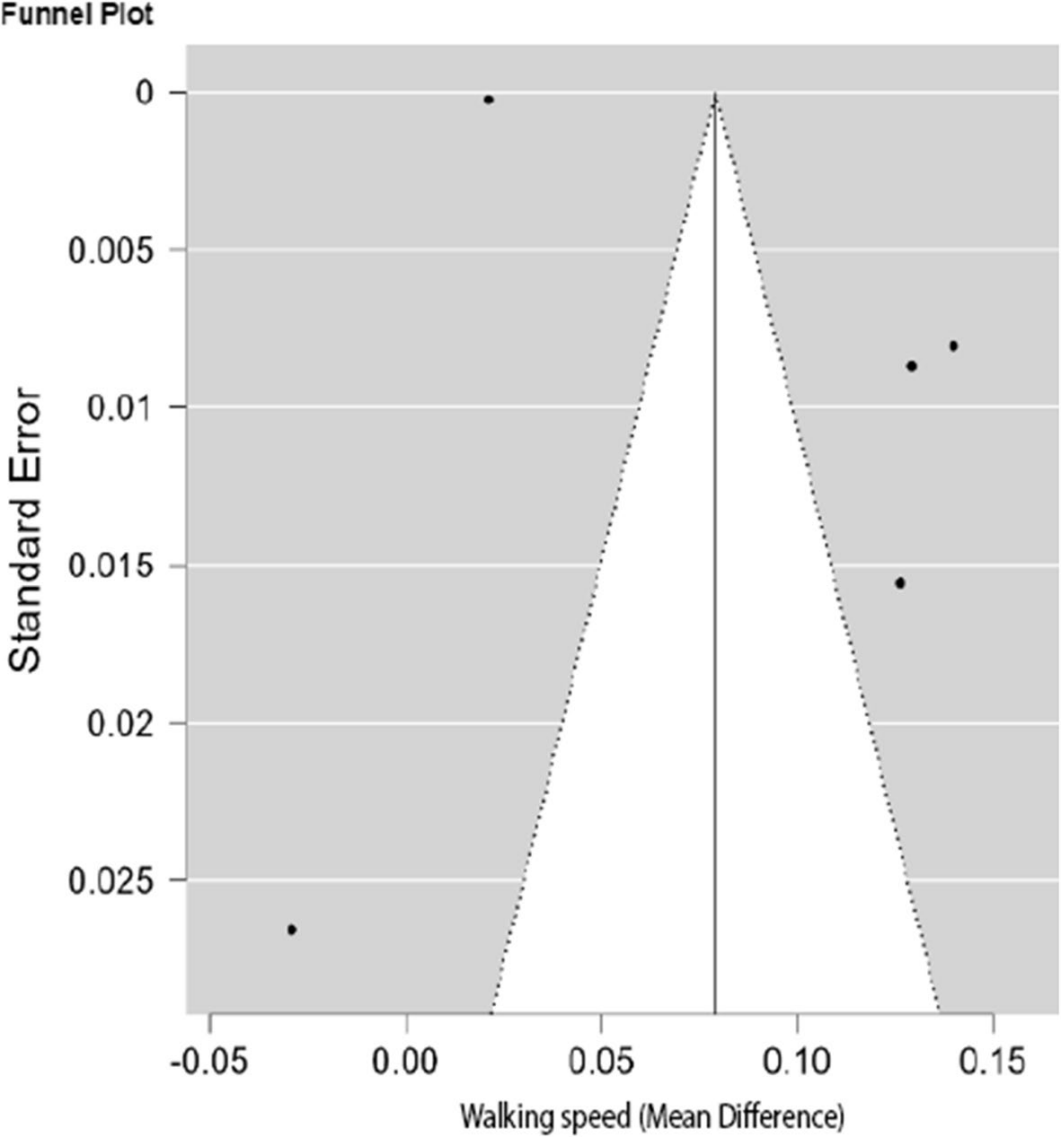
Funnel plot for studies in the meta-analysis of the difference in mean walking speed between participants exposed to anticholinergic medication and those not exposed

**Fig. 3:**
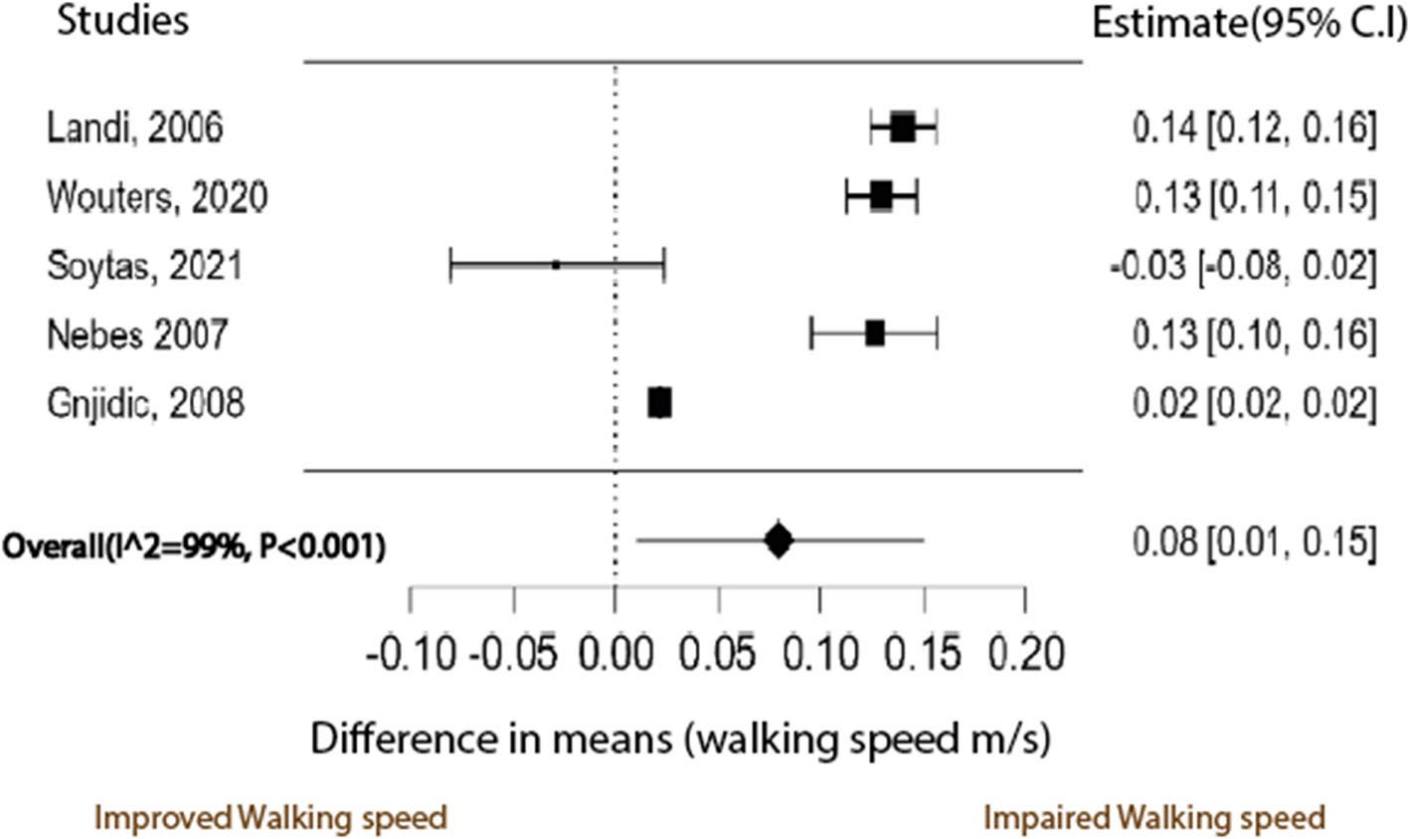
A meta-analysis of the difference in mean walking speeds between participants not exposed to anticholinergic medication and those exposed to anticholinergic medication

Using the DerSimonian and Laird method [27], the random-effects model was used to meta-analyse the data to establish the overall change in the mean difference in walking speed scores between participants exposed to anticholinergic medication and those not exposed to anticholinergic medication. Figure 2 below is a funnel plot of the five studies used in the walking speed meta-analysis. The small number of studies makes visual inspection for funnel plot asymmetry inconclusive. However, as all but one study showed a positive association of AB with walking speed, publication bias is implied. This contrasts with the statistically non-significant Eggers Regression test for funnel plot asymmetry (*P* = 0.494). However, when the number of studies is low, as is the case here, the statistical power of Egger’s test may not be high enough to detect real asymmetry. Therefore, Stern et al. [52] recommend at least n ≥ 10 studies for Eggers regression to be relevant. Figure 3 is a forest plot of the meta-analysis between AB and walking speed. The studies in the walking speed meta-analysis show a high degree of heterogeneity (I^2^ = 99% *p* < 0.01). The instrument for walking speed was the same in all studies, so the difference in means was used as the effect estimate. The pooled estimated effect of AB on mobility was a general reduction in physical function as measured by the walking speed instrument. The pooled estimated effect of AB on walking speed was a decrease of 0.079 m/s ± 0.035 (MD ± SE 95% CI: 0.010 to 0.149) *p* = 0.026. The minimum clinically important difference (MCID) of walking speed, calculated by Bohannon and Glenney [53], is 0.11 m/s. Thus, the pooled estimated effect is less than the MCID and thus considered a small effect size. For this reason, confidence in the evidence was not upgraded. The confidence intervals overlap the MCID, suggesting imprecision of the effect. For this, the certainty of the evidence was downgraded. The results of the univariate meta-regression revealed that part of the variance in the effect size was explained by the choice of AB scale in the studies (*r*^2^ = 0.24) and not by the mean age nor percentage of females in the studies (*r*^2^ = 0).

### Anticholinergic burden and IADL

**Fig. 4:**
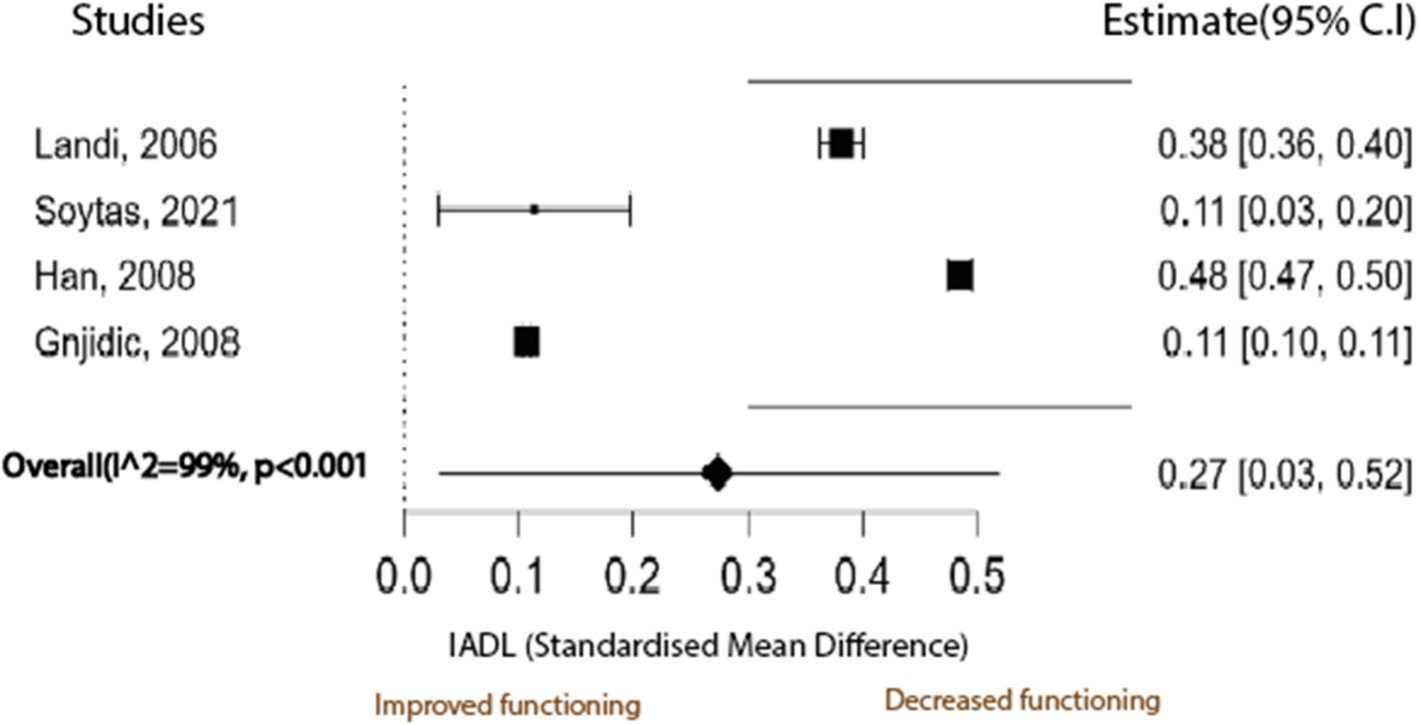
A meta-analysis of the difference in mean IADL scores between participants exposed to anticholinergic medication and those not exposed to anticholinergic medication

The random-effects model was also used to meta-analyse data on the association between AB and IADL scores. Two of the four studies in the meta-analysis used the IADL [33, 36] as developed by Lawton et al. [54], while the other two used modified versions of the IADL score [30, 34]. Ordinarily, higher IADL scores indicate high functioning and, therefore, greater independence, while low IADL scores indicate poor physical functioning and greater dependence. However, two studies [30, 33] used an inverted IADL scale where low IADL scores represent high functioning and vice versa for high IADL scores. Hence, the direction of the effect was used to pool the results for meta-analysis. The mean differences obtained showed that mobility was impaired in all four studies. Landi et al. [30] assessed IADL using the minimum data set for home care (MDS-HC) instrument [55], whilst Han et al. [34] used the IADL scale of the older American resources and services (OARS) instrument [56]. The mean differences were standardised because of the different modifications in the IADL instrument used to measure the underlying singular construct [19]. The studies in this meta-analysis for IADL and AB show a high degree of heterogeneity (I^2^ = 99% *p* < 0.001, Fig. 4). The pooled estimated effect of AB on IADL was a decrease of 0.27 ± 0.12 (SMD ± SE 95% CI: 0.03 to 0.52), *p* = 0.027. Thus, mobility was negatively impaired in both outcomes (IADL scores and walking speed). However, like with walking speed, this change in the IADL score is less than the MCID of 0.47 [57] and has confidence intervals that overlap this MCID. There were not enough studies to perform a univariate meta-regression for the types of AB scales used since all four studies in the meta-analysis used four different AB scales. The meta-regression with mean age and percentage of females showed that these variables did not contribute to the heterogeneity observed (*r*^2^ = 0).

### Studies with a focus on specific anticholinergic drugs

Two studies did not employ an anticholinergic rating scale instead compared taking a single anticholinergic drug with a placebo group [40] or having two groups stratified by the anticholinergic drug they were taking [36]. The study by Moretti et al. [36] had an equal number of participants in group A taking olanzapine *n* = 178 and group B with participants taking either haloperidol or promazine chloridate, both known to have anticholinergic effects [8]. Longitudinal progression in both groups was analysed at baseline and a follow-up after 12 months. The group taking olanzapine showed only moderate worsening of mobility that was not significant *p* > 0.05 in gait, balance and equilibrium (as stated by the Tinetti scale) when following-up results were compared with baseline. However, group B, with participants taking either haloperidol or promazine, registered significantly worsened scores for physical performance in follow-up to baseline (*p* < 0.05 and *p* < 0.01, ΔBI and ΔTinetti, respectively). However, the study by Street et al. [40] demonstrated olanzapine is strongly associated with gait. The placebo group did not receive any anticholinergic medication, whilst three intervention groups had participants taking either 5 mg/d, 10 mg/d or 15 mg/d of the anticholinergic drug olanzapine. All three intervention groups had higher odds of abnormal gait than the placebo group (OR: 11.2, 7.5, 9.4 for 5,10,15 mg/d, respectively).

### Ceiling and floor effects

Three studies discussed the possibility of analysis being affected negatively by a “ceiling” or “floor” effect. To address this concern, Pasina et al. [43] used BI scores to discriminate participation, with those scoring less than 20 at baseline excluded from the study altogether. This was done to exclude patients with the highest degree of physical impairment, in whom it might be more difficult to detect any potential effects of anticholinergic drugs on physical performance. The study by Hilmer et al. [37] also introduced exclusion criteria to avoid the floor effect by requiring that subjects not report difficulty walking 0.25 miles, climbing ten steps, or performing activities of daily living as measured at baseline. On the other hand, the paper by Wilson et al. [39], the only study not to suggest an association between AB and poor physical performance, did not extend the exclusion criterion to filter out participants with already poor physical performance. However, the study suggested that a ceiling effect might have been introduced by a “decline in cholinergic receptors present in the very old populations with high rates of cognitive impairment that is independent of external exposure to anticholinergic medications.”

### Dose–response relationship

Most of the studies only stratified users into either 1) users of anticholinergic drugs and 2) non-users of anticholinergic drugs. This did not permit analysis of a dose–response relationship. Only four studies [11, 30, 31, 33] further stratified the anticholinergic drug user group into moderate and high users. Of the four, only three [11, 30, 33] investigated the relationship between this categorical stepwise dose difference and its impact on walking speed. All three studies observed a dose–response relationship whereby the group with a high anticholinergic burden had a slower walking speed than the group with a moderate anticholinergic burden. This observation increased confidence that an effect existed, and GRADE was upgraded accordingly.

## Discussion

This systematic review is the first to the author’s knowledge to investigate the association between AB and mobility as the primary outcome and explore a dose–response relationship. The studies in this systematic review revealed the importance of stratifying the analysis by age, as the cognitive profiles of people is observed to be age dependent. The review has also revealed the need to capture several mobility measures when conducting studies as some physical impairment measures may take more time than others to manifest.

One study showed for the first time that “young-old” (54-74yrs) and “old-old” participants (≥ 75) users of anticholinergic drugs have distinct cognitive profiles [41]. For example, TUG scores were more frequently impaired in non-users of anticholinergic drugs only in the old-old adults and not the young-old adults. The findings of the same study suggested that anticholinergic drugs mainly affect mobility through cognition in old-old adults; after poor TUG scores lost their association with anticholinergic drug consumption when TMT-B scores (trail making test B-scores) were included in the same multivariate model. The findings support previous studies suggesting a compromised blood–brain barrier permeability that arises with age [58], where BBB permeability was observed to increase. A more permeable BBB will experience a greater cognitive anticholinergic burden. Studies suggest a global association between cognition and mobility which increases with age [59], stating that mobility relies on cognitive processes to anticipate and adapt to the moving environment while maintaining postural control and motor coordination [60].

The studies in this review also highlighted the possible important consideration of ensuring that different aspects of mobility measures are included in a study design to increase the chances that an effect of anticholinergic medication on physical outcomes is revealed if at all it exists; as opposed to having one measure of mobility which might lead to an erroneous suggestion that there is no association. It is also possible that impaired mobility is progressive, with successive stages. That would mean that impairment to one mobility measure might happen sooner than others, whilst another might need a longer time to express itself.

The exposure in this systematic review was the consumption of drugs with anticholinergic properties as defined by several anticholinergic scales. There could have been misclassification of AB exposure because studies have used different AB scales to quantify AB. Also, the exposure group of each study lumped together all participants taking anticholinergic drugs without discriminating on the number of anticholinergic drugs being taken since participants were broadly classified as either users or non-users of anticholinergic drugs. It is, therefore, possible that participants in the exposure group of one study were exposed to more or less AB than the exposure group in other studies, leading to a disparity in observed effects across studies. Thus, the considerable heterogeneity observed (${I}^{2}$=99%) is to an extent expected and could not be avoided in this systematic review. For this reason, confidence in the certainty of the evidence was downgraded by only one point in the GRADE assessment as opposed to the possible two points. Additionally, some heterogeneity may have been introduced by different population characteristics. For example, some of the study participants in one study were characterised by one or more of fourteen clinical conditions such as diabetes, dementia, renal failure and hypertension [30]. Other studies did not capture or detail the clinical conditions of participants. The disparity in the clinical conditions of participants across studies may translate to an inconsistency in observed effects.

Age and multimorbidity are risk factors for polypharmacy. The more morbidities a person has, the more medications they are likely to take and the greater the chances that some of their medication has anticholinergic properties. This would suggest that more unwell/sick participants would be in the group taking anticholinergic medicine than in the group not taking an anticholinergic medicine. This residual confounding would have the effect of diminishing the effect size. For this reason, the confidence in the level of certainty was upgraded to counteract this. The systematic review focused on older people equal to or above 65. Thus, the systematic review indirectly answers the question of whether or not there is an association between anticholinergic burden and mobility, as the results may not be generalized to the entire population. This would have the effect of downgrading the certainty of evidence.

### Strengths and limitations

The heterogeneity of the studies in terms of the AB scales used and choice of mobility outcome measure made it challenging to pool results together in a meta-analysis. Only one study [40] investigated the dose–response relationship between anticholinergic medication and mobility, but mobility was not the primary outcome. There were not enough studies to conduct a meaningful meta-analysis on the effect of low AB on mobility versus the effect of high AB on mobility. In general, the low number of studies available for meta-analysis was a major limitation and suggested the existence of publication bias. However, because Egger’s test did not suggest publication bias, the confidence in the certainty of the evidence was not sufficiently reduced to warrant a downgrade in the GRADE assessment.

For the studies included in the meta-analysis, walking speed and IADL were in the primary outcome group, included in the primary outcome measure of mobility. In addition, the studies included were conducted across several countries, allowing the findings in this study to have greater generalizability.

## Conclusion

This review reveals challenges that can be addressed with future research. For example, we do not understand the complex interaction of duration and severity of AB exposure on mobility outcomes. In particular, there is a lack of information on which types of medicines are strongly associated with impaired mobility. However, the review’s findings suggest a negative association between anticholinergic medicine exposure and mobility, inferred mostly from observational study designs. The causal relationship can only be answered through interventional studies. Though AB is an important prognostic predictor of impaired mobility in older adults, there is very little guidance for clinicians on the suitability of the published scales to measure AB exposure accurately.

## Supplementary Information

**Additional file 1. **Search Strategy.

**Additional file 2. **Modified NOS for assessing the risk of bias for cross-sectional studies.

**Additional file 3. **Cochrane risk of bias tool for randomised trials was used (ROB1).

**Additional file 4. **Summary of Findings (SoF) table, GRADE assessment.

**Additional file 5. **Modified NOS for assessing the risk of bias for cohort studies.

## Data Availability

All data generated or analysed during this study are included in this published article [and its supplementary information files].

## Abbreviations

AA: Anticholinergic activity
AB: Anticholinergic burden
ACB: Anticholinergic cognitive burden
ARS: Anticholinergic risk scale
AEC: Anticholinergic effects on cognitions
BI: Barthel index
TUG: Timed-up and go
SPPB: Short physical performance battery
JASP: Jeffreys’s amazing statistics program
