# Supplementary material for: The association between anticholinergic burden and mobility: a systematic review and meta-analyses
Source: BMC Geriatr. 2023 Mar 22;23:161. doi: 10.1186/s12877-023-03820-6 (PMC10035151; doi:10.1186/s12877-023-03820-6)
Supplement: Supplementary file 1 — Additional file 1. Search Strategy. [file 12877_2023_3820_MOESM1_ESM.docx]

**Additional File 1**

**The association between anticholinergic burden and mobility: A systematic review and meta-analyses**

**preliminary search strategy**

Searches will be conducted in Medline, Embase, PsycINFO, Cochrane CENTRAL and Cinahl using key words in titles and abstracts, as well as MeSH terms. For each database, the search terms will address three themes; 1) the exposure, 2)outcome and 3)population of interest. Thus the terms anticholinergic, mobility and older adults will be used with their synonyms respectively. The following table shows how the searches will be conducted in Medline and Embase databases. Only studies that divided participants into users and non users of anticholinergic drugs passed the prescreen and were discussed in the results section.

| **Database** | **Search**  **#** | **Search** **query** | **Results** |
| --- | --- | --- | --- |
| **Medline**  **(PubMed)** | #1 | (((anticholinergic[MeSH Major Topic]) OR antimuscarinic) OR Acetylcholine Antagonist[MeSH Major Topic]) OR Cholinergic Antagonists | 46, 856 |
|  | #2 | ((mobility[MeSH Major Topic]) OR gait) OR falls | 43, 8749 |
|  | #3 | (((Geriatric) OR older adult) OR elderly) OR old | 3, 836, 204 |
|  | **#4** | **#1** **AND** **#2** **AND** **#3**  ((((((Geriatric) OR older adult) OR elderly) OR old)) AND (((mobility[MeSH Major Topic]) OR gait) OR falls)) AND ((((anticholinergic[MeSH Major Topic]) OR antimuscarinic) OR Acetylcholine Antagonist[MeSH Major Topic]) OR Cholinergic Antagonists) | 1,164 |
| **Embase** | #1 | ('cholinergic receptor blocking agent')/br OR (('muscarinic receptor blocking agent')/exp) OR (('anticholinergic effect')/exp) | 358, 014 |
|  | #2 | ('joint mobility')/br OR (('walking speed')/exp) OR ((falls)/exp) | 42, 047 |
|  | #3 | ('older adults')/br OR ((geriatrics)/exp) OR ((aged)/exp) | 3, 663, 227 |
|  | #4 | **#1** **AND** **#2** **AND** **#3** | 77 |
