# Supplementary material for: The association between anticholinergic burden and mobility: a systematic review and meta-analyses
Source: BMC Geriatr. 2023 Mar 22;23:161. doi: 10.1186/s12877-023-03820-6 (PMC10035151; doi:10.1186/s12877-023-03820-6)
Supplement: Supplementary file 2 — Additional file 2. Modified NOS for assessing the risk of bias for cross-sectional studies. [file 12877_2023_3820_MOESM2_ESM.docx]

**MODIFIED NEWCASTLE - OTTAWA QUALITY ASSESSMENT SCALE: Adapted for Cross-sectional Studies**

**Author**: Mayer et al., 2017 [12]

**Title:** Comparison of Nine Instruments to Calculate Anticholinergic Load in a Large Cohort of Older Outpatients: Association with Cognitive and Functional Decline, Falls, and Use of Laxatives

Note: A study can be awarded a maximum of one star for each numbered item, except for 1) Ascertainment of exposure, 2)Comparability and 3)Assessment of outcome; which can have a maximum of two stars.

| No. | Criterion | Decision rule | Score (*=1, no*=0) | Location in text |
| --- | --- | --- | --- | --- |
| SELECTION (Max 5 stars) | | | | |
| 1 | Representativeness of the sample | a)Truly representative of the elderly population e.g random sampling * (SELECTED)  b)Somewhat representative of the elderly population (non-random sampling) *  b) Selected group of users  c) No description of the sampling strategy | 1 | **METHODS**  **Study Sample**  Home-dwelling  participants (N = 2,761) aged between 60 and 87 years |
| 2 | Selected group of users | a) Systematic selection of participants e.g  -Are taking anticholinergic drugs for the same reason  -All have same diagnosis e.g same disease/condition.  This helps exclude certain factors such as disease variability which may bias results *  b)Not relevant systematic selection (SELECTED) | 0 |  |
| 3 | Sample size | a)Justified and satisfactory (power calculation included) *  b)Not justified | 0 |  |
| 4 | Ascertainment of exposure | a)Validated measuring tool used and description of medicine reconciliation for non-SAA scales **(SELECTED)  b)Validated measuring tool used and but no description of medicine reconciliation (for non-SAA scales) *  c)Non-validated measurement tool, but the tool is available or described, description of medicine reconciliation for non-SAA scales **  d)Non-validated measurement tool, but the tool is available or described, no description of medicine reconciliation for non-SAA scales *  e)No description of the measurement tool | 2 | **METHODS**  **Calculating the Anticholinergic Load and**  **Defining Anticholinergic Drug Users**  The remaining eight scales11–18 and the DBI19 were  applied to calculate the participant’s individual anticholinergic  load. Therefore, the scale-based instruments  sum up the score of each identified anticholinergic drug  whereas the DBI takes into account the daily taken dose  and the minimum recommended daily dose of the respective  drug |
| COMPARABILITY (Maximum 2 stars) | | | | |
| 5 | The subjects in the two outcome groups are comparable. Confounding factors are controlled for | a)Study controls for age*  b)Study controls for sex, age, performance .** (SELECTED)  c)Limited or no attempt to control for differences between the cohorts | 2 | **METHODS**  **Statistical Analysis**  All multivariate  analyses were adjusted for age, sex, family  status, BMI, CrCI, CIRS-G SI, and CFS. |
| OUTCOME (Maximum 3 stars) | | | | |
| 6 | Assessment of outcome | a)Independent or blind assessment stated, or confirmation of the outcome by reference to secure records **  b)record linkage* (SELECTED)  c) Clinical assessment, no description of blinding*  d)Self-reported  e)No description | 1 | Reference 7  **MATERIALS AND METHODS**  **Study Population**  Our data originate from three different sources: self-reported information from a participant questionnaire, information on the participant from the corresponding GP, and information collected by a study physician visiting the participant at home for geriatric assessment. |
| 7 | Statistical Test | a)The statistical test used to analyze the data is clearly described and appropriate, and the measurement of the association is presented e.g confidence intervals and the probability level (p value) * (SELECTED)  b)The statistical test is not appropriate, not described or incomplete | 1 | **METHODS**  **Statistical Analysis**  To characterize the study cohort, means with standard  deviations (SDs) and median with interquartile  ranges (IQRs) were calculated for continuous data. For  categorical variables, absolute numbers and the percentage  proportions are reported. |
|  |  | SCORE: | 7/10 | |

**MODIFIED NEWCASTLE - OTTAWA QUALITY ASSESSMENT SCALE: Adapted for Cross-sectional Studies**

**Author**: Attoh-Mensah et al., 2020 [14] **Title:** Anticholinergic Drug Use and Negative Outcomes Among the Frail Elderly Population Living in a Nursing Home

Note: A study can be awarded a maximum of one star for each numbered item, except for 1) Ascertainment of exposure, 2)Comparability and 3)Assessment of outcome; which can have a maximum of two stars.

| No. | Criterion | Decision rule | Score (*=1, no*=0) | Location in text |
| --- | --- | --- | --- | --- |
| SELECTION (Max 5 stars) | | | | |
| 1 | Representativeness of the sample | a)Truly representative of the elderly population e.g random sampling *  b)Somewhat representative of the elderly population (non-random sampling) * (SELECTED)  b) Selected group of users  c) No description of the sampling strategy | 1 | **ABSTRACT**  **Objectives**  age groups (“young–old”  55–74 vs. “old–old” ≥ 75 years).  **METHODS**  **Participants**  They were  recruited over a 5-year period, between late 2011 and early  2017, among community-dwelling older adults. |
| 2 | Selected group of users | a) Systematic selection of participants e.g  -Are taking anticholinergic drugs for the same reason  -All have same diagnosis e.g same disease/condition.  This helps exclude certain factors such as disease variability which may bias results * (SELECTED)  b)Not relevant systematic selection | 1 | **METHODS**  **Participants**  All participants had experienced one or more fall, with  or without injury, in the year before the study. |
| 3 | Sample size | a)Justified and satisfactory (e.g power calculation included) * (SELECTED)  b)Not justified | 1 | **Study Limitations**  Second, the  relatively small sample size is inherent to the design of  the study, in which only volunteers who agreed to attend  for cognitive and mobility assessments could be included.  However, the small sample size, which limits statistical  power, can be viewed as a strength when compared  with epidemiological studies in which some outcomes  of relevance cannot be obtained. |
| 4 | Ascertainment of exposure | a)Validated measuring tool used and description of medicine reconciliation for non-SAA scales ** (SELECTED)  b)Validated measuring tool used and but no description of medicine reconciliation (for non-SAA scales) *  c)Non-validated measurement tool, but the tool is available or described, description of medicine reconciliation for non-SAA scales **  d)Non-validated measurement tool, but the tool is available or described, no description of medicine reconciliation for non-SAA scales *  e)No description of the measurement tool | 2 | **METHODS**  **Anticholinergic Drug Identification**  Drugs with anticholinergic (antimuscarinic) properties were identified using the Anticholinergic Drug Scale (ADS)  The number of anticholinergic drugs used  daily and the anticholinergic cumulative burden was then determined for each participant |
| COMPARABILITY (Maximum 2 stars) | | | | |
| 5 | The subjects in the two outcome groups are comparable. Confounding factors are controlled for | a)Study controls for age*  b)Study controls for sex, age, performance .**  c)Limited or no attempt to control for differences between the cohorts | 2 | **METHODS**  **Statistical Analysis**  The adjustment variables  were age, educational level, BMI, handgrip strength,  risk factors for falls, and comorbidities, as appropriate. Only  the adjusted variables with a regression p value < 0.15 |
| OUTCOME (Maximum 3 stars) | | | | |
| 6 | Assessment of outcome | a)Independent or blind assessment stated, or confirmation of the outcome by reference to secure records ** (SELECTED)  b)record linkage*  c) Clinical assessment, no description of blinding*  d)Self-reported  e)No description | 2 | **METHODS**  **Outcome Measures**  Trained neuropsychologists who  were blinded to the participants’ medical treatments evaluated  cognition and mobility. |
| 7 | Statistical Test | a)The statistical test used to analyze the data is clearly described and appropriate, and the measurement of the association is presented e.g confidence intervals and the probability level (p value) *(SELECTED)  b)The statistical test is not appropriate, not described or incomplete | 1 | METHODS  Statistical Analysis  We compared the characteristics of users and nonusers of anticholinergic drugs, by age group, using Student’s twotailed  t test, the Mann–Whitney U test, or the Chi-squared test, as appropriate |
|  |  | SCORE: | 10/10 | |

**MODIFIED NEWCASTLE - OTTAWA QUALITY ASSESSMENT SCALE: Adapted for Cross-sectional Studies**

**Author**: Landi et al., 2006 [16] **Title:** Anticholinergic Drugs and Physical Function Among Frail Elderly Population

Note: A study can be awarded a maximum of one star for each numbered item, except for 1) Ascertainment of exposure, 2)Comparability and 3)Assessment of outcome; which can have a maximum of two stars.

| No. | Criterion | Decision rule | Score (*=1, no*=0) | Location in text |
| --- | --- | --- | --- | --- |
| SELECTION (Max 5 stars) | | | | |
| 1 | Representativeness of the sample | a)Truly representative of the elderly population e.g random sampling * (SELECTED)  b)Somewhat representative of the elderly population (non-random sampling) *  b) Selected group of users  c) No description of the sampling strategy | 1 | **ABSTRACT**  status in persons aged 80 years or older. Data are from baseline evaluation of 364 subjects enrolled in the ilSIRENTE  study. The ilSIRENTE study is a prospective cohort study performed in the mountain community living in the Sirente  geographic area (L’Aquila, Abruzzo) in Central Italy |
| 2 | Selected group of users | a) Systematic selection of participants e.g  -Are taking anticholinergic drugs for the same reason  -All have same diagnosis e.g same disease/condition.  This helps exclude certain factors such as disease variability which may bias results *  b)Not relevant systematic selection (SELECTED) | 0 |  |
| 3 | Sample size | a)Justified and satisfactory (e.g power calculation included) *  b)Not justified (SELECTED) | 0 |  |
| 4 | Ascertainment of exposure | a)Validated measuring tool used and description of medicine reconciliation for non-SAA scales ** (SELECTED)  b)Validated measuring tool used and but no description of medicine reconciliation (for non-SAA scales) *  c)Non-validated measurement tool, but the tool is available or described, description of medicine reconciliation for non-SAA scales **  d)Non-validated measurement tool, but the tool is available or described, no description of medicine reconciliation for non-SAA scales *  e)No description of the measurement tool | 2 | **ABSTRACT**  We defined as anticholinergic drugs all  medications for which serum anticholinergic activity was previously demonstrated. |
| COMPARABILITY (Maximum 2 stars) | | | | |
| 5 | The subjects in the two outcome groups are comparable. Confounding factors are controlled for | a)Study controls for age*  b)Study controls for sex, age, performance .** (SELECTED)  c)Limited or no attempt to control for differences between the cohorts | 2 | **METHODS**  **Statistical Analysis**  Final analyses were adjusted for age, gender, smoking,  physical activity level, dementia, living alone, BMI, congestive heart  failure, lung diseases, and diabetes. The cognitive performance scale  score was excluded from the |
| OUTCOME (Maximum 3 stars) | | | | |
| 6 | Assessment of outcome | a)Independent or blind assessment stated, or confirmation of the outcome by reference to secure records **  b)record linkage* (SELECTED)  c) Clinical assessment, no description of blinding*  d)Self-reported  e)No description | 1 | **METHODS**  **Data Collection**  Clinical diagnoses were recorded by study physicians based on  information collected from the patient and the general practitioner  on physical examination, careful review of patient clinical  documentation (including, lab tests and X-rays), and previous  medical history. |
| 7 | Statistical Test | a)The statistical test used to analyze the data is clearly described and appropriate, and the measurement of the association is presented e.g confidence intervals and the probability level (p value) * (SELECTED)  b)The statistical test is not appropriate, not described or incomplete | 1 | METHODS  Statistical Analysis  Baseline characteristics of the study sample  between anticholinergic drug users and non-users were compared  using analysis of variance analyses for normally distributed variables,  non-parametric Kruskal–Wallis H-tests for skewed variables, and w2  analyses for dichotomous variables. |
|  |  | SCORE: | 7/10 | |

**MODIFIED NEWCASTLE - OTTAWA QUALITY ASSESSMENT SCALE: Adapted for Cross-sectional Studies**

**Author**: Pasina et al., 2013 [17] **Title:** Anticholinergic Drug Use and Negative Outcomes Among the Frail Elderly Population Living in a Nursing Home

Note: A study can be awarded a maximum of one star for each numbered item, except for 1)Ascertainment of exposure, 2)Comparability and 3)Assessment of outcome; which can have a maximum of two stars.

| No. | Criterion | Decision rule | Score (*=1, no*=0) | Location in text |
| --- | --- | --- | --- | --- |
| SELECTION (Max 5 stars) | | | | |
| 1 | Representativeness of the sample | a)Truly representative of the elderly population e.g random sampling *  b)Somewhat representative of the elderly population (non-random sampling) * (SELECTED)  b) Selected group of users  c) No description of the sampling strategy | 1 | Reference 30  **ABSTRACT**  **Methods**  This cross-sectional study was held in 38 Italian internal medicine and geriatric wards participating in the Registro Politerapie SIMI (REPOSI) study during 2008. The study sample included 1155 in-patients aged 65 years or older. |
| 2 | Selected group of users | a) Systematic selection of participants e.g  -Are taking anticholinergic drugs for the same reason  -All have same diagnosis e.g same disease/condition.  This helps exclude certain factors such as disease variability which may bias results * (SELECTED)  b)Not relevant systematic selection | 1 | Reference 30  **Materials and Methods**  **Data Collection**  The specific aims of the REPOSI study were: to describe the prevalence of concurrent multiple diseases and treatments in elderly in-patients; to correlate their clinical characteristics with the type and number of diseases and treatments and to examine the main clinical outcomes at hospital discharge |
| 3 | Sample size | a)Justified and satisfactory (e.g power calculation included) * (SELECTED)  b)Not justified | 1 | **Reference 30**  **Strengths and Limitations**  The major strengths of the REPOSI study include the multicenter design that involved 38 internal medicine and geriatric wards throughout Italy, resulting in a sample representative of the hospitalized elderly population; |
| 4 | Ascertainment of exposure | a)Validated measuring tool used and description of medicine reconciliation for non-SAA scales ** (SELECTED)  b)Validated measuring tool used and but no description of medicine reconciliation (for non-SAA scales) *  c)Non-validated measurement tool, but the tool is available or described, description of medicine reconciliation for non-SAA scales **  d)Non-validated measurement tool, but the tool is available or described, no description of medicine reconciliation for non-SAA scales *  e)No description of the measurement tool | 2 | **METHODS**  **Exposure to Anticholinergic Drugs**  For the purposes of this study, we calculated the drug related anticholinergic burden for each patient, using the  sum of the points for each anticholinergic medication dispensed at hospital admission, according to both scales. |
| COMPARABILITY (Maximum 2 stars) | | | | |
| 5 | The subjects in the two outcome groups are comparable. Confounding factors are controlled for | a)Study controls for age*  b)Study controls for sex, age, performance .** (SELECTED)  c)Limited or no attempt to control for differences between the cohorts | 2 | METHODS  Statistical Analysis  Multivariate analyses of the  association between SBT and anticholinergic drug use were  adjusted for the known risk factors such as age, sex, education,  and history of stroke or transient ischaemic attack (TIA) and number of non anticholinergic drugs as possible  confounders, while analyses for the association between anticholinergic drug use and BI were adjusted for age, sex and  CIRS severity index. |
| OUTCOME (Maximum 3 stars) | | | | |
| 6 | Assessment of outcome | a)Independent or blind assessment stated, or confirmation of the outcome by reference to secure records **  b)record linkage* (SELECTED)  c) Clinical assessment, no description of blinding*  d)Self-reported  e)No description | 1 | METHODS  Data collection  The REPOSI study is a collaborative, independent, voluntary  effort by the Italian Society of InternalMedicine (SIMI) and the  Mario Negri Institute for Pharmacological Research, and has  been described elsewhere in detail |
| 7 | Statistical Test | a)The statistical test used to analyze the data is clearly described and appropriate, and the measurement of the association is presented e.g confidence intervals and the probability level (p value) * (SELECTED)  b)The statistical test is not appropriate, not described or incomplete | 1 | METHODS  Statistical Analysis  The Pearson correlation coefficient of the univariate model was used to study the association between the change in the BI  score and change in ACB or ARS scores. Analyses were done  with JMP Pro 9 (SAS Institute Inc., Cary, NC, USA). |
|  |  | SCORE: | 9/10 | |

**MODIFIED NEWCASTLE - OTTAWA QUALITY ASSESSMENT SCALE: Adapted for Cross-sectional Studies**

**Author**: Soytas et al., 2021 [22] **Title:** Association between anticholinergic drug burden with sarcopenia, anthropometric measurements, and comprehensive geriatric assessment

parameters in older adults Note: A study can be awarded a maximum of one star for each numbered item, except for 1) Ascertainment of exposure, 2)Comparability and 3)Assessment of outcome; which can have a maximum of two stars.

| No. | Criterion | Decision rule | Score (*=1, no*=0) | Location in text |
| --- | --- | --- | --- | --- |
| SELECTION (Max 5 stars) | | | | |
| 1 | Representativeness of the sample | a)Truly representative of the elderly population e.g random sampling *  b)Somewhat representative of the elderly population (non-random sampling) * (SELECTED)  b) Selected group of users  c) No description of the sampling strategy | 1 | **METHODS**  **Study Population and Design**  The population of this single-center, cross-sectional study consists of  volunteer patients 65 years and over who applied to the geriatrics  outpatient clinic between January 2019 and March 2020. |
| 2 | Selected group of users | a) Systematic selection of participants e.g  -Are taking anticholinergic drugs for the same reason  -All have same diagnosis e.g same disease/condition.  This helps exclude certain factors such as disease variability which may bias results * (SELECTED)  b)Not relevant systematic selection | 1 | **METHODS**  **Study Population and Design**  Patients with  cognitive dysfunction (advanced dementia, delirium, permanent  cognitive dysfunction due to a previous cerebrovascular accident) who  could not comply with comprehensive geriatric assessment (CGA) tests  were excluded from the study. |
| 3 | Sample size | a)Justified and satisfactory (e.g power calculation included) *  b)Not justified (SELECTED) | 0 |  |
| 4 | Ascertainment of exposure | a)Validated measuring tool used and description of medicine reconciliation for non-SAA scales ** (SELECTED)  b)Validated measuring tool used and but no description of medicine reconciliation (for non-SAA scales) *  c)Non-validated measurement tool, but the tool is available or described, description of medicine reconciliation for non-SAA scales **  d)Non-validated measurement tool, but the tool is available or described, no description of medicine reconciliation for non-SAA scales *  e)No description of the measurement tool | 2 | **METHODS**  **Calculation of anticholinergic burden**  The ACB scale was used to calculate the ADB. Drugs scored 0 do not  have anticholinergic effects. |
| COMPARABILITY (Maximum 2 stars) | | | | |
| 5 | The subjects in the two outcome groups are comparable. Confounding factors are controlled for | a)Study controls for age*  b)Study controls for sex, age, performance .**  c)Limited or no attempt to control for differences between the cohorts (SELECTED) | 0 |  |
| OUTCOME (Maximum 3 stars) | | | | |
| 6 | Assessment of outcome | a)Independent or blind assessment stated, or confirmation of the outcome by reference to secure records **  b)record linkage*  c) Clinical assessment, no description of blinding*  d)Self-reported  e)No description | 0 |  |
| 7 | Statistical Test | a)The statistical test used to analyze the data is clearly described and appropriate, and the measurement of the association is presented e.g confidence intervals and the probability level (p value) * (SELECTED)  b)The statistical test is not appropriate, not described or incomplete | 1 | **METHODS**  **Statistical Analysis**  Correlation analysis between ADB, demographic  data, anthropometric measurements, CGA parameters, and  sarcopenia parameters was conducted by the Spearman method. Age,  mid-upper circumference, calf circumference, HGS, MMSE, BADLs were  analyzed with Linear Regression (LR) method to evaluate the most  relevant parameters with the anticholinergic drug burden. p-value of  <0.05 was considered statistically significant. |
|  |  | SCORE: | 5/10 | |

**MODIFIED NEWCASTLE - OTTAWA QUALITY ASSESSMENT SCALE: Adapted for Cross-sectional Studies**

**Author**: Nebes et al., 2007 [23] **Title:** Serum Anticholinergic Activity and Motor Performance in Elderly Persons.

Note: A study can be awarded a maximum of one star for each numbered item, except for 1) Ascertainment of exposure, 2)Comparability and 3)Assessment of outcome; which can have a maximum of two stars.

| No. | Criterion | Decision rule | Score (*=1, no*=0) | Location in text |
| --- | --- | --- | --- | --- |
| SELECTION (Max 5 stars) | | | | |
| 1 | Representativeness of the sample | a)Truly representative of the elderly population e.g random sampling *  b)Somewhat representative of the elderly population (non-random sampling) * (SELECTED)  b) Selected group of users  c) No description of the sampling strategy | 1 | **INTRODUCTION**  The present study examined whether an elevated anticholinergic  burden in high-functioning community volunteers is  associated with decrements on tests of motor performance  (gait speed and simple manual response time) known to  predict falls in elderly persons |
| 2 | Selected group of users | a) Systematic selection of participants e.g  -Are taking anticholinergic drugs for the same reason  -All have same diagnosis e.g same disease/condition.  This helps exclude certain factors such as disease variability which may bias results * (SELECTED)  b)Not relevant systematic selection | 1 | **METHODS**  **Participants**  To exclude cognitively-impaired individuals, potential participants were  given the Repeatable Battery for the Assessment of Neuropsychological  Status (8). Of 90 individuals tested, two had an age-adjusted scaled score on this battery more than 1.5  standard deviations below the mean and so were excluded. |
| 3 | Sample size | a)Justified and satisfactory (e.g power calculation included) *  b)Not justified | 0 |  |
| 4 | Ascertainment of exposure | a)Validated measuring tool used and description of medicine reconciliation for non-SAA scales ** (SELECTED)  b)Validated measuring tool used and but no description of medicine reconciliation (for non-SAA scales) *  c)Non-validated measurement tool, but the tool is available or described, description of medicine reconciliation for non-SAA scales **  d)Non-validated measurement tool, but the tool is available or described, no description of medicine reconciliation for non-SAA scales *  e)No description of the measurement tool | 2 | **METHODS**  **Procedure**  Prior to testing, 10 cc of blood were drawn to measure  SAA using a radioreceptor competitive binding assay  Another potential confounder is participant health. In particular,  individuals with cerebrovascular disease often have  psychomotor deficits and may coincidently be taking many  medications, including anticholinergics (e.g., digoxin).  Thus, it could be a difference in neurological status and  not SAA that produced the performance differences between  groups. To estimate the severity of cerebrovascular disease  in our participants, we created a Framingham Stroke Risk  Profile for each participant (12). |
| COMPARABILITY (Maximum 2 stars) | | | | |
| 5 | The subjects in the two outcome groups are comparable. Confounding factors are controlled for | a)Study controls for age*  b)Study controls for sex, age, performance .** (SELECTED)  c)Limited or no attempt to control for differences between the cohorts | 2 | **METHODS**  **Procedure**  Even after controlling for age and  sex, SAA group still had a significant effect on the two  performance tasks (F ¼ 3.43, df ¼ 4,164; p ¼ .01). |
| OUTCOME (Maximum 3 stars) | | | | |
| 6 | Assessment of outcome | a)Independent or blind assessment stated, or confirmation of the outcome by reference to secure records **  b)record linkage*  c) Clinical assessment, no description of blinding* (SELECTED)  d)Self-reported  e)No description | 1 | **METHODS**  **Procedure**  Participants walked a 15-foot course along a carpeted  corridor. They began from a full stop and were told to walk  at their usual pace until they crossed the marked finish line.  Walking time was measured with a stopwatch to the nearest  1/10th of a second from the signal to begin until the participant’s  foot crossed the 15-foot mark…. |
| 7 | Statistical Test | a)The statistical test used to analyze the data is clearly described and appropriate, and the measurement of the association is presented e.g confidence intervals and the probability level (p value) * (SELECTED)  b)The statistical test is not appropriate, not described or incomplete | 1 | **RESULTS**  Univariate analyses of variance were run between the  three SAA groups on participant characteristics and performance  measures |
|  |  | SCORE: | 8/10 | |

**MODIFIED NEWCASTLE - OTTAWA QUALITY ASSESSMENT SCALE: Adapted for Cross-sectional Studies**

**Author**: Gnjidic et al., 2008 [26] **Title:** Drug Burden Index and physical function in older Australian men

Note: A study can be awarded a maximum of one star for each numbered item, except for 1) Ascertainment of exposure, 2)Comparability and 3)Assessment of outcome; which can have a maximum of two stars.

| No. | Criterion | Decision rule | Score (*=1, no*=0) | Location in text |
| --- | --- | --- | --- | --- |
| SELECTION (Max 5 stars) | | | | |
| 1 | Representativeness of the sample | a)Truly representative of the elderly population e.g random sampling * (SELECTED)  b)Somewhat representative of the elderly population (non-random sampling) *  b) Selected group of users  c) No description of the sampling strategy | 1 | **INTRODUCTION**  The aim of this study  was to evaluate the association between the Drug Burden  Index and physical performance and functional status  measures in a random sample of community-dwelling  older men,aged70 years,  **ABSTRACT**  **Methods**  A cross-sectional survey was performed on community-dwelling older  men enrolled in The Concord Health and Ageing in Men Project,  Sydney, Australia.  **METHODS**  Study Population  The only exclusion criterion was living  in a residential aged care facility. |
| 2 | Selected group of users | a) Systematic selection of participants e.g  -Are taking anticholinergic drugs for the same reason  -All have same diagnosis e.g same disease/condition.  This helps exclude certain factors such as disease variability which may bias results * (SELECTED)  b)Not relevant systematic selection | 1 | **METHODS**  Study Population  The only exclusion criterion was living  in a residential aged care facility. |
| 3 | Sample size | a)Justified and satisfactory (e.g power calculation included) * (SELECTED)  b)Not justified | 1 | **DISCUSSION**  The CHAMP population represents a large random  sample of community-dwelling older Australian men. |
| 4 | Ascertainment of exposure | a)Validated measuring tool used and description of medicine reconciliation for non-SAA scales ** (SELECTED)  b)Validated measuring tool used and but no description of medicine reconciliation (for non-SAA scales) *  c)Non-validated measurement tool, but the tool is available or described, description of medicine reconciliation for non-SAA scales **  d)Non-validated measurement tool, but the tool is available or described, no description of medicine reconciliation for non-SAA scales *  e)No description of the measurement tool | 2 | METHODS  Medication Exposure  Information about medication use was collected during the clinic visit. Participants were instructed to bring all prescription and over-the-counter medications with them to the clinic visit.  Use of sedative and anticholinergic medications was  quantified using the Drug Burden Index, a measure of a  person’s exposure to medications with anticholinergic and  sedative properties that incorporates the principles of  dose–response and maximal effect |
| COMPARABILITY (Maximum 2 stars) | | | | |
| 5 | The subjects in the two outcome groups are comparable. Confounding factors are controlled for | a)Study controls for age*  b)Study controls for sex, age, performance .** (SELECTED)  c)Limited or no attempt to control for differences between the cohorts | 2 | ABSTRACT  Results  After adjusting for confounders(sociodemographics, comorbidities, cognitive impairment, depression), |
| OUTCOME (Maximum 3 stars) | | | | |
| 6 | Assessment of outcome | a)Independent or blind assessment stated, or confirmation of the outcome by reference to secure records **  b)record linkage*  c) Clinical assessment, no description of blinding* (SELECTED)  d)Self-reported  e)No description | 1 | **METHODS**  **Outcome measures**  Physical performance was assessed by administering the  performance battery, a modification of the Established  Populations for Epidemiological Studies of the Elderly  summary performance score. |
| 7 | Statistical Test | a)The statistical test used to analyze the data is clearly described and appropriate, and the measurement of the association is presented e.g confidence intervals and the probability level (p value) * (SELECTED)  b)The statistical test is not appropriate, not described or incomplete | 1 | **METHODS**  **Statistical Analysis**  All analyses were performed with SAS statistical software (version 9.1; SAS Institute,Cary, NC, USA). All tests were two  tailed. Statistical significance was set at P < 0.05. |
|  |  | SCORE: | 9/10 | |

**MODIFIED NEWCASTLE - OTTAWA QUALITY ASSESSMENT SCALE: Adapted for Cross-sectional Studies**

**Author**: Cao et al., 2008 [27] **Title:** Anticholinergic Drug Use and Negative Outcomes Among the Frail Elderly Population Living in a Nursing Home

Note: A study can be awarded a maximum of one star for each numbered item, except for 1) Ascertainment of exposure, 2)Comparability and 3)Assessment of outcome; which can have a maximum of two stars.

| No. | Criterion | Decision rule | Score (*=1, no*=0) | Location in text |
| --- | --- | --- | --- | --- |
| SELECTION (Max 5 stars) | | | | |
| 1 | Representativeness of the sample | a)Truly representative of the elderly population e.g random sampling *  b)Somewhat representative of the elderly population (non-random sampling) * (SELECTED)  b) Selected group of users  c) No description of the sampling strategy | 1 | **Abstract**  The study population comprised 932 moderately to severely disabled community resident women aged 65 years or older who were participants in the Women's Health and Aging Study I. |
| 2 | Selected group of users | a) Systematic selection of participants e.g  -Are taking anticholinergic drugs for the same reason  -All have same diagnosis e.g same disease/condition.  This helps exclude certain factors such as disease variability which may bias results * (SELECTED)  b)Not relevant systematic selection | 1 | **Abstract**  The study population comprised 932 moderately to severely disabled community resident women aged 65 years or older who were participants in the Women's Health and Aging Study I. |
| 3 | Sample size | a)Justified and satisfactory (e.g power calculation included) *  b)Not justified (SELECTED) | 0 |  |
| 4 | Ascertainment of exposure | a)Validated measuring tool used and description of medicine reconciliation for non-SAA scales **  b)Validated measuring tool used and but no description of medicine reconciliation (for non-SAA scales) * (SELECTED)  c)Non-validated measurement tool, but the tool is available or described, description of medicine reconciliation for non-SAA scales **  d)Non-validated measurement tool, but the tool is available or described, no description of medicine reconciliation for non-SAA scales *  e)No description of the measurement tool | 1 | **Abstract**  To test this hypothesis, we developed a continuous scale to quantify drug burden and used this scale to analyze cross-sectional physical function data from the Women’s Health and Aging Study I (WHAS I), carried out in Baltimore, MD, from 1992 to 1994.  **Discussion**  There is a lack of drug exposure/effect information over a broad range of exposure, making imprecise the characterization of the drug exposure  below which there is no observable effect and above which there is no additional effect. |
| COMPARABILITY (Maximum 2 stars) | | | | |
| 5 | The subjects in the two outcome groups are comparable. Confounding factors are controlled for | a)Study controls for age*  b)Study controls for sex, age, performance .** (SELECTED)  c)Limited or no attempt to control for differences between the cohorts | 2 | **METHODS**  **Statistical analysis**  We adjusted for the effect of age, race, education, depressive symptoms, arthritis,  self-reported visual impairment, self-reported hearing impairment, hypertension, ischemic heart disease, congestive heart failure, pulmonary disease, osteoporosis, diabetes mellitus, cancer, disc disease, hip fracture, spinal stenosis, Parkinson’s disease, and  peripheral arterial disease, as they were likely to be associated with  functional outcomes |
| OUTCOME (Maximum 3 stars) | | | | |
| 6 | Assessment of outcome | a)Independent or blind assessment stated, or confirmation of the outcome by reference to secure records **  b)record linkage*  c) Clinical assessment, no description of blinding* (SELECTED)  d)Self-reported  e)No description | 1 | **METHODS**  **Data Collection**  The data for this analysis come from the screening and baseline interviews and examination, all conducted in the  participants’ homes.28,29 The interview included standardized questions about physician-diagnosed chronic diseases and difficulty with upper extremity functioning, mobility, and ADLs. In addition, the Geriatric Depression Scale30 and the MMSE31 were administered. The examination included standardized blood pressure and joint examination, a series of anthropometric measurements, and physical performance tests such as grip strength, usual walking speed over 4 m, progressive standing balance (side by side, semi tandem, and full tandem—each for 10 s), and repeated chair stands. |
| 7 | Statistical Test | a)The statistical test used to analyze the data is clearly described and appropriate, and the measurement of the association is presented e.g confidence intervals and the probability level (p value) *  b)The statistical test is not appropriate, not described or incomplete | 1 | **RESULTS**  **Association between drug burden and physical function**  The odds ratio (OR) for balance difficulty in anticholinergic  drug users reached 4.9 with 95% confidence interval  (CI) 2.0–12.0 (Po0.0005) |
|  |  | SCORE: | 7/10 | |
