# Supplementary material for: The association between anticholinergic burden and mobility: a systematic review and meta-analyses
Source: BMC Geriatr. 2023 Mar 22;23:161. doi: 10.1186/s12877-023-03820-6 (PMC10035151; doi:10.1186/s12877-023-03820-6)
Supplement: Supplementary file 3 — Additional file 3. Cochrane risk of bias tool for randomised trials was used (ROB1). [file 12877_2023_3820_MOESM3_ESM.docx]

The Cochrane Collaboration’s Tool For Assessing Risk Of Bias

**Author:** Kolanowski, 2015 [13] **Title:** Anticholinergic Exposure During Rehabilitation: Cognitive and Physical Function Outcomes in Patients with Delirium Superimposed on Dementia

| Domain | Support for judgement | Review authors’ judgement |
| --- | --- | --- |
| *Selection Bias* | | |
| **Random sequence generation** | Not stated | Unclear |
| **Allocation concealment** | (ClinicalTrials.gov identifier: NCTO1267682).  **METHODS**  **Participants**  Randomization was concealed until after enrollment and was conducted using SAS Release 9.3 using randomly permuted blocks of sizes 2, 4 and 6 to ensure approximately balanced intervention group sizes across the length of the study, and to control for possible temporal effects. | **Low risk of bias** |
| *Performance bias* | | |
| **Blinding participants and personnel:** Assessments are made for each main outcome (or class of outcomes) | (ClinicalTrials.gov identifier: NCTO1267682).  **METHODS**  **Participants**  All outcomes were measured by trained assessors, blind to randomization. We maintained blinding by keeping assessment and intervention teams separate in the clinical area and during research team meetings | **Low risk of bias**, although single blind |
| *Detection bias* | | |
| **Blinding of outcome assessment:** Assessments are made for each main outcome (or class of outcomes) | (ClinicalTrials.gov identifier: NCTO1267682).  **METHODS**  **Participants**  All outcomes were measured by trained assessors, blind to randomization. We maintained blinding by keeping assessment and intervention teams separate in the clinical area and during research team meetings  **Statistical Analysis**  The intention-to-treat principle was used for analysis, and the statistician was blind to group assignment until all analyses were complete | **Low risk of bias** |
| *Attrition bias* | | |
| **Incomplete outcome data:** Assessments are made for each main outcome (or class of outcomes) | (ClinicalTrials.gov identifier: NCTO1267682).  **Statistical Analysis**  Power to detect a difference in mean levels of severity and duration of delirium between the groups was estimated a priori, assuming a total sample size of 256 participants after attrition, and adjusting for cluster effects due to multiple observations made on the same subjects. | **Low risk of bias** |
| *Reporting bias* | | |
| **Selective reporting** | (ClinicalTrials.gov identifier: NCTO1267682).  **RESULTS**  Completion of outcome assessments was excellent. We obtained 92.6% and 95.3% of all possible CAM and DRS assessments; 86.3% and 79.1% of all possible MoCA and CLOX assessments; and 95.8% of all possible BI assessments. There were no differences in missing data between the groups, and we found no evidence that missing data were due to any substantive correlations with demographic, or clinical characteristics. | **Low risk of bias** |
| *Other bias* | | |
| **Other sources of bias** | **METHODS**  **Setting and Sample**  The presence of  delirium was established by screening potential  participants using two instruments: 1) the Mini-  Mental State Exam,16 a 30-item cognitive screen,  and 2) the Confusion Assessment Method,17 a standardized  diagnostic algorithm for delirium. | **Low risk of bias**, similar baseline characteristics |

**Author:** Wilson, 2010 [18] **Title:** Associations between drug burden index and physical function in older people in residential aged care facilities

| Domain | Support for judgement | Review authors’ judgement |
| --- | --- | --- |
| *Selection Bias* | | |
| **Random sequence generation** | NCT00322166 at ClinicalTrials.gov  **METHODS**  **Randomisation and intervention**  The random allocation sequence, which was in permuted blocks of size 6 or 3, was generated by a statistician who was not involved in the recruitment; and it was concealed from the study coordinators until after randomisation. | **Low risk of bias** |
| **Allocation concealment** | NCT00322166 at ClinicalTrials.gov  **METHODS**  **Randomisation and intervention**  The random allocation sequence, which was in permuted blocks of size 6 or 3, was generated by a statistician who was not involved in the recruitment; and it was concealed from the study coordinators until after randomisation. | **Low risk of bias** |
| *Performance bias* | | |
| **Blinding participants and personnel:** Assessments are made for each main outcome (or class of outcomes) | NCT00322166 at ClinicalTrials.gov  **DISCUSSION**  There was no blinding among participants or research staff, although laboratory staff conducting the biochemical analyses were unaware of group assignment and falls were documented by facility staff who were usually unaware of the resident being part of a study. | **High risk of bias,** no blinding does not account for possible placebo effect. |
| *Detection bias* | | |
| **Blinding of outcome assessment:** Assessments are made for each main outcome (or class of outcomes) | NCT00322166 at ClinicalTrials.gov  **DISCUSSION**  There was no blinding among participants or research staff, although laboratory staff conducting the biochemical analyses were unaware of group assignment and falls were documented by facility staff who were usually unaware of the resident being part of a study. | **Low risk of bias,** laboratory staff (assessors) were unaware of group assignment. |
| *Attrition bias* | | |
| **Incomplete outcome data:** Assessments are made for each main outcome (or class of outcomes) | NCT00322166 at ClinicalTrials.gov  **RESULTS**  **Adherence**  Despite the appointment of Sunlight Officers, adherence to increased sunlight exposure was generally low: 70% of the 397 subjects attended >10% of the sessions, 44% attended >30% of the sessions and only 17% of subjects attended >50% of the available 260 sessions in a year. The rate of dropout was reasonably constant over the study period and by season. | **High risk of bias**, adherence rate dropped to 46% by end of study |
| *Reporting bias* | | |
| **Selective reporting** | No description | Unclear |
| *Other bias* | | |
| **Other sources of bias** | NCT00322166 at ClinicalTrials.gov  **RESULTS**  **Falls**  We adjusted for these and other variables previously shown to predict falls [33], namely age, sex, past falls, urinary incontinence, cognitive impairment, static balance, co-morbidities, care level (RCS) and GDS | **Low risk of bias,** differences in other characteristics was accounted for |

**Author:** Street, 2000 [21] **Title:** Olanzapine Treatment of Psychotic and Behavioral Symptoms in Patients With Alzheimer Disease in Nursing Care Facilities: A double-blind , Randomised, Placebo-Controlled Trial

| Domain | Support for judgement | Review authors’ judgement |
| --- | --- | --- |
| *Selection Bias* | | |
| **Random sequence generation** | PATIENTS AND METHODS  Study Design  Patients meeting enrolment criteria were randomly allocated to 1 of 4 fixed-dose treatment groups (Olanzapine, 5, 10, 15 mg/d, or placebo) by the assignment of a unique kit number using a permuted block design at each investigational site (block size of 4) | Low risk of bias |
| **Allocation concealment** | No description | Unclear risk of bias |
| *Performance bias* | | |
| **Blinding participants and personnel:** Assessments are made for each main outcome (or class of outcomes) | ABSTRACT  Methods  A multicentre, double-blind, placebo-controlled, 6-week study was conducted in 206 elderly US nursing home residents with AD | **Low risk of bias,** although the use of the term “double-blind” is not explicitly described |
| *Detection bias* | | |
| **Blinding of outcome assessment:** Assessments are made for each main outcome (or class of outcomes) | PATIENTS AND METHODS  Assessments  All patient assessments were conducted at the nursing facility by health care professionals, including neurologists, psychiatrists, geriatricians, psychometrists, nurses, and other medical specialists trained before study initiation | **Unclear risk of bias**, does not mention whether the assessors where blinded/unaware of allocation |
| *Attrition bias* | | |
| **Incomplete outcome data:** Assessments are made for each main outcome (or class of outcomes) | RESULTS  Efficacy Results  Figure 1 | **Low risk of bias,** the attrition rate was below 40% |
| *Reporting bias* | | |
| **Selective reporting** | RESULTS  Safety results  Table 3: Data are presented as number (percentage) and include all treatment-emergent adverse events with an incidence ≥10% or significantly greater than placebo, regardless of cause | **Low risk of bias** |
| *Other bias* | | |
| **Other sources of bias** | No description | Unclear risk of bias |
