# Supplementary material for: The association between anticholinergic burden and mobility: a systematic review and meta-analyses
Source: BMC Geriatr. 2023 Mar 22;23:161. doi: 10.1186/s12877-023-03820-6 (PMC10035151; doi:10.1186/s12877-023-03820-6)
Supplement: Supplementary file 4 — Additional file 4. Summary of Findings (SoF) table, GRADE assessment. [file 12877_2023_3820_MOESM4_ESM.docx]

| Question: What is the association between anticholinergic burden and mobility? | | | | | | | | | |
| --- | --- | --- | --- | --- | --- | --- | --- | --- | --- |
| **Quality Assessment**  **A mix of RCT’s & Observational studies, evidence commenced as moderate** | | | | | | | | |  |
| **Studies** | **Risk of bias** | **Inconsistency/**  **Heterogeneity** | **Indirectness** | **Imprecision** | **Publication bias** | **Large Magnitude of effect** | **Dose-response** | **Effect of all plausible confounding factors** | **Overall quality of evidence** |
| 3 RCT’s  13 Observational  Studies  TOTAL: 16 | No serious concern | Some concern ↓ | Indirectness present↓ | Imprecision observed ↓ | No serious concern | Small magnitude | Dose-response observed↑ | Diminishes observed effect size↑ | LOW  ⊕⊕⊖⊖ |

Summary of findings (SoF) table, GRADE approach
