# Supplementary material for: The association between anticholinergic burden and mobility: a systematic review and meta-analyses
Source: BMC Geriatr. 2023 Mar 22;23:161. doi: 10.1186/s12877-023-03820-6 (PMC10035151; doi:10.1186/s12877-023-03820-6)
Supplement: Supplementary file 5 — Additional file 5. Modified NOS for assessing the risk of bias for cohort studies. [file 12877_2023_3820_MOESM5_ESM.docx]

**MODIFIED NEWCASTLE - OTTAWA QUALITY ASSESSMENT SCALE: COHORT STUDIES**

**Author**: Landi et al., 2014 **Title:** Anticholinergic Drug Use and Negative Outcomes Among the Frail Elderly Population Living in a Nursing Home

Note: A study can be awarded a maximum of one star for each numbered item within the Selection and Outcome categories. A maximum of two stars can be given for Comparability

| No. | Criterion | Decision rule | Score (*=1, no*=0) | Location in text |
| --- | --- | --- | --- | --- |
| SELECTION | | | | |
| 1 | Representativeness of the exposed cohort | a)Truly representative of the elderly population (at least 65 years old) * (SELECTED)  b)Subgroup of the exposed population are elderly (at least 65 years old) or all participants would be ≥65 by end of study *  b) Not satisfying requirements in part (a) or (b), or not stated | 1 | **Methods:** The only exclusion criterion was age younger than 65 years |
| 2 | Selection of the non-exposed cohort | a)Selected from the same source population as exposed cohort * (SELECTED)  b)Selected from a different population  c) No description | 1 | **Results:** Main characteristics of the sample population stratified according  to anticholinergic drug use are shown in Table 2. |
| 3 | Ascertainment of exposure | a)Record completed by medical staff * (SELECTED)  b)Structured interview*  c)Written self-report  d) No description | 1 | **METHODS, Anticholinergic Drugs:** Trained study personnel collected detailed information about  medication use during nursing home assessment using a structured  questionnaire embedded in the MDS-NH form. The study personnel  collected information on drugs received by each resident in the  7 days preceding the baseline assessment. Drugs were coded  according to the Anatomical Therapeutic and Chemical codes |
| 4 | Demonstration that outcome of interest was not present at the start of the study | a)Yes*  b)No or not explicitly stated (SELECTED) | 0 | Methods: The only exclusion criterion was age younger than 65 years |
| COMPARABILITY | | | | |
| 5 | Comparability of cohorts on the basis of the design or analysis | a)Study controls for age*  b)Study controls for sex, age, performance at baseline**  c)Limited or no attempt to control for differences between the cohorts (SELECTED) | 0 |  |
| OUTCOME | | | | |
| 6 | Assessment of outcome | a)Independent or blind assessment stated, or confirmation of the outcome by reference to secure records*  b)record linkage*  c)Self-reported  d)No description | 1 | **Methods:** We used data from the database of the U.L.I.S.S.E project (Un Link Informatico sui Servizi  Sanitari Esistenti per l’Anziani), a prospective multicenter observational study. |
| 7 | Was follow-up long enough for outcomes to occur? | a)Yes* (SELECTED)  b)No  c)Length of follow up not stated | 1 | **Methods:** Patients from 31 facilities  in Italy were assessed at baseline and at 6 and 12 months by trained personnel |
| 8 | Adequacy of follow up of cohorts | a)Complete follow up-all participants accounted for * (SELECTED)  b) Subjects lost to follow up unlikely to introduce bias (<20% lost to follow up, or description provided of those lost) *  c)Follow up rate <85% and no description of those lost provided  d)No statement | 1 | **Table 2:** Sociodemographic and Clinical Characteristics of Study Participants According to  Anticholinergic Drug Use (ARS) (Individuals With Follow-up 1490) |
|  |  | SCORE: | 6 | |

**MODIFIED NEWCASTLE - OTTAWA QUALITY ASSESSMENT SCALE: COHORT STUDIES**

**Author**: Pasina et al., 2013 **Title:** Association of Anticholinergic Burden with Cognitive and Functional Status in a Cohort of Hospitalized Elderly: Comparison of the Anticholinergic Cognitive Burden Scale and Anticholinergic Risk Scale

Note: A study can be awarded a maximum of one star for each numbered item within the Selection and Outcome categories. A maximum of two stars can be given for Comparability

| No. | Criterion | Decision rule | Score (*=1, no*=0) | Location in text |
| --- | --- | --- | --- | --- |
| SELECTION | | | | |
| 1 | Representativeness of the exposed cohort | a)Truly representative of the elderly population (at least 65 years old) * (SELECTED)  b)Subgroup of the exposed population are elderly (at least 65 years old) or all participants would be ≥65 by end of study *  b) Not satisfying requirements in part (a) or (b), or not stated | 1 | **Methods:** The sample included 1,380  inpatients aged 65 years or older. |
| 2 | Selection of the non-exposed cohort | a)Selected from the same source population as exposed cohort *  b)Selected from a different population  c) No description | 1 | **Methods:** This cross-sectional, prospective study (3-month  telephone follow-up) was conducted in 66 Italian internal  medicine and geriatric wards participating in the Registry of  Polytherapies SIMI |
| 3 | Ascertainment of exposure | a)Record completed by medical staff * (SELECTED)  b)Structured interview*  c)Written self-report  d) No description | 1 | **Methods, Exposure to Anticholinergic Drugs:** For the purposes of this study, we calculated the drugrelated  anticholinergic burden for each patient, using the  sum of the points for each anticholinergic medication dispensed  at hospital admission, according to both scales. |
| 4 | Demonstration that outcome of interest was not present at the start of the study | a)Yes* (SELECTED)  b)No or not explicitly stated | 1 | **Methods:** In order to avoid ceiling effects, we excluded any patients with a score lower than 15, so as to exclude most patients with the highest degree of physical impairment, in whom it might be more difficult to detect  any potential effects of anticholinergic drugs on the basic  activities of daily living. |
| COMPARABILITY | | | | |
| 5 | Comparability of cohorts on the basis of the design or analysis | a)Study controls for age*  b)Study controls for sex, age, performance at baseline** (SELECTED)  c)Limited or no attempt to control for differences between the cohorts | 2 | **Methods,**  *Statistical Analysis:*  Multivariate analyses of the  association between SBT and anticholinergic drug use were  adjusted for the known risk factors such as age, sex, education,  and history of stroke or transient ischaemic attack  (TIA) and number of non-anticholinergic drugs as possible  confounders, while analyses for the association between anticholinergic drug use and BI were adjusted for age, sex and  CIRS severity index. |
| OUTCOME | | | | |
| 6 | Assessment of outcome | a)Independent or blind assessment stated, or confirmation of the outcome by reference to secure records* (SELECTED)  b)record linkage*  c)Self-reported  d)No description | 1 | **Methods,**  *Data Collection*  The attending physicians completed  a standardized web-based Case Report Form 2 days of hospital admission or when patients were considered  stable. |
| 7 | Was follow-up long enough for outcomes to occur? | a)Yes* (SELECTED)  b)No  c)Length of follow up not stated | 1 | **Methods,**This cross-sectional, prospective study (3-month  telephone follow-up) |
| 8 | Adequacy of follow up of cohorts | a)Complete follow up-all participants accounted for *  b) Subjects lost to follow up unlikely to introduce bias (<20% lost to follow up, or description provided of those lost) *  c)Follow up rate <85% and no description of those lost provided  d)No statement (SELECTED) | 0 |  |
|  |  | SCORE: | 8 | |

**MODIFIED NEWCASTLE - OTTAWA QUALITY ASSESSMENT SCALE: COHORT STUDIES**

**Author**: Wouters et al., 2020 **Title:** Long-Term Exposure to Anticholinergic and Sedative Medications and Cognitive and Physical Function in Later Life

Note: A study can be awarded a maximum of one star for each numbered item within the Selection and Outcome categories. A maximum of two stars can be given for Comparability

| No. | Criterion | Decision rule | Score (*=1, no*=0) | Location in text |
| --- | --- | --- | --- | --- |
| SELECTION | | | | |
| 1 | Representativeness of the exposed cohort | a)Truly representative of the elderly population (at least 65 years old) *  b)Subgroup of the exposed population are elderly (at least 65 years old) or all participants would be ≥65 by end of study * (SELECTED)  b) Not satisfying requirements in part (a) or (b), or not stated | 1 | **Methods**  *Participants and Study Design*  Participants were aged 55–85 years at baseline in 1992/1993. |
| 2 | Selection of the non-exposed cohort | a)Selected from the same source population as exposed cohort * (SELECTED)  b)Selected from a different population  c) No description | 1 | **Methods,**  *Participant and Study Design*  The sample was recruited from registries of 11 municipalities  in three geographic regions in The Netherlands. |
| 3 | Ascertainment of exposure | a)Record completed by medical staff *  b)Structured interview* (SELECTED)  c)Written self-report  d) No description | 1 | **Methods,**  *Cumulative Exposure to Anticholinergic and Sedative Medications*  As part of the medical interview conducted at each measurement  occasion, participants were asked to show their medication containers.  The name, dose, frequency of intake, and duration of use of  every medication was recorded on a standardized form |
| 4 | Demonstration that outcome of interest was not present at the start of the study | a)Yes* (SELECTED)  b)No or not explicitly stated | 1 | **Methods,**  *Participants and Study Design*  For the present  analyses, we excluded participants with potential drinking problems  in the past and present (ie, ≥6 glasses of alcohol at least once a week  or 21 days per month drinking ≥4 glasses), and those who reported  to have severe hearing and vision problems. This was done, because  excessive alcohol consumption and sensory deficits are likely to  bias performance on tests of cognitive and physical functioning |
| COMPARABILITY | | | | |
| 5 | Comparability of cohorts on the basis of the design or analysis | a)Study controls for age*  b)Study controls for sex, age, performance at baseline** (SELECTED)  c)Limited or no attempt to control for differences between the cohorts | 2 | **Methods,**  *Statistical Analysis*  Analyses were adjusted for sex, education  (years), age, living with a partner, BMI, depressive symptoms, number  of comorbidities, and prescribed medications |
| OUTCOME | | | | |
| 6 | Assessment of outcome | a)Independent or blind assessment stated, or confirmation of the outcome by reference to secure records* (SELECTED)  b)record linkage*  c)Self-reported  d)No description | 1 | **Methods,**  *Participants and Study Design*  Data were collected by trained interviewers in participants’ homes  through a main interview lasting on average 1 hour and 45 minutes,  a self-report questionnaire, and an additional medical interview |
| 7 | Was follow-up long enough for outcomes to occur? | a)Yes* (SELECTED)  b)No  c)Length of follow up not stated | 1 | **Methods:** Older adult participants of the Longitudinal Aging Study Amsterdam (LASA) were followed from 1992 to 2012. |
| 8 | Adequacy of follow up of cohorts | a)Complete follow up-all participants accounted for *  b) Subjects lost to follow up unlikely to introduce bias (<20% lost to follow up, or description provided of those lost) * (SELECTED)  c)Follow up rate <85% and no description of those lost provided  d)No statement | 1 | **Results**: Of the 3,107 individuals who consented to participate, 291 were  excluded because they had no medication use reported and 189  were excluded for other reasons, leaving 2,627 participants eligible  at baseline. A total of 2,252 participants completed the first followup  and 726 completed the final sixth follow-up 20 years later |
|  |  | SCORE: | 9 | |

**MODIFIED NEWCASTLE - OTTAWA QUALITY ASSESSMENT SCALE: COHORT STUDIES**

**Author**: Han et al., 2008 **Title:** Cumulative Anticholinergic Exposure Is Associated with Poor Memory and Executive Function in Older Men

Note: A study can be awarded a maximum of one star for each numbered item within the Selection and Outcome categories. A maximum of two stars can be given for Comparability

| No. | Criterion | Decision rule | Score (*=1, no*=0) | Location in text |
| --- | --- | --- | --- | --- |
| SELECTION | | | | |
| 1 | Representativeness of the exposed cohort | a)Truly representative of the elderly population (at least 65 years old) * (SELECTED)  b)Subgroup of the exposed population are elderly (at least 65 years old) or all participants would be ≥65 by end of study *  b) Not satisfying requirements in part (a) or (b), or not stated | 1 | **PARTICIPANTS:** Five hundred forty-four community dwelling men  aged 65 and older with diagnosed hypertension. |
| 2 | Selection of the non-exposed cohort | a)Selected from the same source population as exposed cohort * (SELECTED)  b)Selected from a different population  c) No description | 1 | **METHODS,**  *Participants*  The study used data from the Connecticut Veterans Longitudinal Cohort, consisting of 767 veterans aged 65 and older recruited at a Department of Veterans Affairs (VA)  primary care clinic between July 2000 and August 2001 |
| 3 | Ascertainment of exposure | a)Record completed by medical staff * (SELECTED)  b)Structured interview*  c)Written self-report  d) No description | 1 | **METHODS,**  *Measure of Anticholinergic and Other Medications*  To apply the clinician-rated anticholinergic scale, two authors (JA and LH) reviewed a complete list of the generic medications used in the current study cohort. |
| 4 | Demonstration that outcome of interest was not present at the start of the study | a)Yes* (SELECTED)  b)No or not explicitly stated | 1 | **METHODS,**  *Outcome Measures*  Trained research personnel administered  the two tests following a standardized protocol, first at baseline  and then at 1- and 2-year follow-up. |
| COMPARABILITY | | | | |
| 5 | Comparability of cohorts on the basis of the design or analysis | a)Study controls for age*  b)Study controls for sex, age, performance at baseline** (SELECTED)  c)Limited or no attempt to control for differences between the cohorts | 2 | **DISCUSSION,**  This association  remained statistically significant after control for other potential  risk factors for cognitive and functional impairment,  including age, education, ADL function, comorbidities,  severity of hypertension, and concomitant use of non-anticholinergic  medications, including antihypertensives and  psychotropics. |
| OUTCOME | | | | |
| 6 | Assessment of outcome | a)Independent or blind assessment stated, or confirmation of the outcome by reference to secure records* (SELECTED)  b)record linkage*  c)Self-reported  d)No description | 1 | **METHODS,**  *Outcome Measures*  Trained research personnel administered  the two tests following a standardized protocol, first at baseline  and then at 1- and 2-year follow-up. |
| 7 | Was follow-up long enough for outcomes to occur? | a)Yes*(SELECTED)  b)No  c)Length of follow up not stated | 1 | **METHODS,**  *Participants*  For each 3-month quarter  over the 2-year follow-up period, a research assistant with  medical training reviewed the primary care visits of these  participants with hypertension. |
| 8 | Adequacy of follow up of cohorts | a)Complete follow up-all participants accounted for *  b) Subjects lost to follow up unlikely to introduce bias (<20% lost to follow up, or description provided of those lost) * (SELECTED)  c)Follow up rate <85% and no description of those lost provided  d)No statement | 1 | **RESULTS,**  Thirteen (2.4%) and 19 (3.6%) participants died during  the first or second year of follow-up, respectively |
|  |  | SCORE: | 9 | |

**MODIFIED NEWCASTLE - OTTAWA QUALITY ASSESSMENT SCALE: COHORT STUDIES**

**Author**: Hilmer et al., 2009 **Title:** Drug Burden Index Score and Functional Decline in Older People

Note: A study can be awarded a maximum of one star for each numbered item within the Selection and Outcome categories. A maximum of two stars can be given for Comparability

| No. | Criterion | Decision rule | Score (*=1, no*=0) | Location in text |
| --- | --- | --- | --- | --- |
| SELECTION | | | | |
| 1 | Representativeness of the exposed cohort | a)Truly representative of the elderly population (at least 65 years old) * (SELECTED)  b)Subgroup of the exposed population are elderly (at least 65 years old) or all participants would be ≥65 by end of study *  b) Not satisfying requirements in part (a) or (b), or not stated | 1 | **Methods,**  *Study Population*  The Health ABC study population consists of 3075 community-resident Medicare recipients aged 70-79 years |
| 2 | Selection of the non-exposed cohort | a)Selected from the same source population as exposed cohort * (SELECTED)  b)Selected from a different population  c) No description | 1 | **Methods,**  *Study Population*  Recruited from April 1997 to June 1998 from areas around Pittsburgh,  Pennsylvania and Memphis, Tennessee. |
| 3 | Ascertainment of exposure | a)Record completed by medical staff * (SELECTED)  b)Structured interview*  c)Written self-report  d) No description | 1 | **Methods,**  *Medical inventory*  A medication inventory was conducted by research personnel during the baseline clinic visit (year 1) and at years 3 and 5. Participants were instructed to bring all prescription and over the counter medications used in the past two weeks to their clinic visit. Staff administered a  structured medication history to confirm medications actually taken by participants in the previous two weeks. |
| 4 | Demonstration that outcome of interest was not present at the start of the study | a)Yes*(SELECTED)  b)No or not explicitly stated | 1 | **Methods,**  *Study Population*  To participate, subjects were required to report no difficulty in walking 0.25 miles, climbing 10 steps, or performing activities of daily living at baseline. |
| COMPARABILITY | | | | |
| 5 | Comparability of cohorts on the basis of the design or analysis | a)Study controls for age*  b)Study controls for sex, age, performance at baseline** (SELECTED)  c)Limited or no attempt to control for differences between the cohorts | 2 | **Figure 1**  DBI and AUCDB grouped into 0, 0-1 and ≥ 1. Means adjusted for year 1 functional score, co-morbidities, hospitalizations and sociodemographic factors using analysis of co-variance. |
| OUTCOME | | | | |
| 6 | Assessment of outcome | a)Independent or blind assessment stated, or confirmation of the outcome by reference to secure records*  b)record linkage*  c)Self-reported  d)No description | 1 | **Methods,**  *Outcome measures*  The primary functional outcome was the short physical performance  battery (SPPB) score, which was obtained at years 1 and 6 of the study. |
| 7 | Was follow-up long enough for outcomes to occur? | a)Yes*(SELECTED)  b)No  c)Length of follow up not stated | 1 | **Discussion**  This longitudinal analysis was limited to the 71% of subjects who had performance data  after **five years of follow-up** and the longitudinal association between DBI and functional  limitation in the excluded population remains unknown |
| 8 | Adequacy of follow up of cohorts | a)Complete follow up-all participants accounted for *  b) Subjects lost to follow up unlikely to introduce bias (<20% lost to follow up, or description provided of those lost) * (SELECTED)  c)Follow up rate <85% and no description of those lost provided  d)No statement | 1 | **Discussion**  This longitudinal analysis was limited to the **71% of subjects** who had performance data  after five years of follow-up and the longitudinal association between DBI and functional  limitation in the excluded population remains unknown |
|  |  | SCORE: | 9 | |
